# *Brucella melitensis* Wzm/Wzt System: Changes in the Bacterial Envelope Lead to Improved Rev1Δ*wzm* Vaccine Properties

**DOI:** 10.3389/fmicb.2022.908495

**Published:** 2022-07-04

**Authors:** Sara Mena-Bueno, Irati Poveda-Urkixo, Oihane Irazoki, Leyre Palacios, Felipe Cava, Ana Zabalza-Baranguá, María Jesús Grilló

**Affiliations:** ^1^Animal Health Department, Instituto de Agrobiotecnología (IdAB, CSIC-Gobierno de Navarra), Pamplona, Spain; ^2^Agronomy, Biotecnology and Food Department, Universidad Pública de Navarra (UPNA), Pamplona, Spain; ^3^Laboratory for Molecular Infection Medicine Sweden, Department of Molecular Biology, Umeå Centre for Microbial Research, Umeå University, Umeå, Sweden

**Keywords:** lipopolysaccharide, Wzm/Wzt system, Rev1Δ*wzm* vaccine, *Brucella* envelope, pregnant sheep, pregnant mice

## Abstract

The lipopolysaccharide (LPS) O-polysaccharide (O-PS) is the main virulence factor in *Brucella*. After synthesis in the cytoplasmic membrane, O-PS is exported to the periplasm by the Wzm/Wzt system, where it is assembled into a LPS. This translocation also engages a bactoprenol carrier required for further biosynthesis pathways, such as cell wall biogenesis. Targeting O-PS export by blockage holds great potential for vaccine development, but little is known about the biological implications of each Wzm/Wzt moiety. To improve this knowledge and to elucidate its potential application as a vaccine, we constructed and studied *wzm*/*wzt* single- and double-deletion mutants, using the attenuated strain *Brucella melitensis* Rev1 as the parental strain. This allowed us to describe the composition of *Brucella* peptidoglycan for the first time. We observed that these mutants lack external O-PS yet trigger changes in genetic transcription and in phenotypic properties associated with the outer membrane and cell wall. The three mutants are highly attenuated; unexpectedly, Rev1Δ*wzm* also excels as an immunogenic and effective vaccine against *B. melitensis* and *Brucella ovis* in mice, revealing that low persistence is not at odds with efficacy. Rev1Δ*wzm* is attenuated in BeWo trophoblasts, does not infect mouse placentas, and is safe in pregnant ewes. Overall, these attributes and the minimal serological interference induced in sheep make Rev1Δ*wzm* a highly promising vaccine candidate.

## Introduction

Brucellosis is one of the most relevant zoonosis worldwide and is caused by bacteria of the genus *Brucella*. These pathogens infect a wide range of domestic and wild animals. *Brucella melitensis* infects predominantly small ruminants and is also the most frequent *Brucella* species in humans in endemic regions. To date, there are no safe vaccines for humans, and antibiotic treatments are onerous with frequent relapses so that the most rational strategy is to control and eradicate animal infections (Blasco, 1997). In small ruminants, Rev1 is the only vaccine recommended (OIE, 2018). However, although attenuated, Rev1 can be pathogenic for animals (for instance, it induces abortions in pregnant ewes; Blasco, 1997) and can infect humans (Spink et al., 1962; Blasco and Díaz, 1993). Thus, finding a safer alternative vaccine is a priority worldwide.^1^ To this end, much research effort has focused on lipopolysaccharide (LPS) modifications and, more specifically, on removing the *N*-formyl-perosamine homopolymer O-polysaccharide (O-PS) (Zhao et al., 2018). Besides being a main virulence factor, O-PS is the immunodominant antigen in *Brucella* (Spink and Anderson, 1954), which is necessary to elicit a protective adaptive immune-response (Montaraz et al., 1986; Grilló et al., 2006b).

Pathways involved in O-PS biosynthesis have been explored as potential targets for vaccine developments (Whitfield, 1995; Schurig et al., 2002; Moriyón et al., 2004; González et al., 2008). In *Brucella*, this molecule is formed in the inner side of the cytoplasmic membrane. It is then translocated to the periplasm by an ATP-binding cassette (ABC) transport system that comprises two essential proteins, Wzt and Wzm, whereby the hydrophilic ATP-binding Wzt is coupled by a unique interface (Bi et al., 2018) to the transmembrane ring-shaped Wzm (Cuthbertson et al., 2007; Mohammad et al., 2016; Caffalette and Zimmer, 2021). This system is broadly conserved among gram-negative bacteria (Whitfield, 1995; Lerouge et al., 2001; Hug and Feldman, 2011; Caffalette et al., 2020), whereby Wzm is strongly conserved, while Wzt has a C-terminal domain (C-Wzt), with a unique structural element that determines the specificity of the O-PS transporter (Izquierdo et al., 2003; Cuthbertson et al., 2005, 2007). Truncation of *wzm/wzt* genes leads to rough (R) mutants carrying O-PS molecules unlinked to the R-LPS rather than smooth (S)-LPS; however, little is known about the effect of each component on *Brucella*. O-PS export further involves the undecaprenol pyro-phosphate (also known as bactoprenol), which is a universal lipid carrier to which sugar precursors attach to initiate O-PS synthesis and export the whole complex to the periplasm (Valvano, 2003). Once O-PS is linked to the LPS, the bactoprenol needs to be released back to the inner membrane, where it can participate other polymerization pathways, such as peptidoglycan (PG) recycling (Valvano, 2015; Vassen, 2018). Thus, we hypothesized that *wzm*/*wzt* truncation and blockage of the O-PS export could alter other bacterial structures and/or functions.

Most *B. melitensis* R mutants were developed from the 16M or H38 virulent strain (Godfroid et al., 2000; González et al., 2008; Wang Z. et al., 2014). However, the background of the parental strain can be crucial for the biological properties of the R derivatives (Barrio et al., 2009). In this work, we built the *wzm*/*wzt* single- and double-mutants from a *B. melitensis* Rev1 attenuated strain, with the objective of understanding how to increase the structural and functional impact derived from disrupting the O-PS export. For this, we analyzed transcriptional changes and features associated with envelope remodeling of Rev1 Wzm/Wzt mutants vs. 16MΔ*wzm* (Zabalza Baranguá, 2017), using state-of-the-art techniques, including transmission electron microscopy (TEM) and ultra-performance liquid chromatography-mass spectrometry (UPLC-MS), as well as *in vivo* experiments in laboratory animals (mice) and in the natural host most susceptible to *B. melitensis* infection (pregnant ewes).

## Results and Discussion

### Deletion of *wzm* and/or *wzt* Induces *wbkB* and *wbkC* Transcriptional Upregulation, but Only Rev1Δ*wzm* Induces a Marked *wzt* Upregulation

**FIGURE 1:**
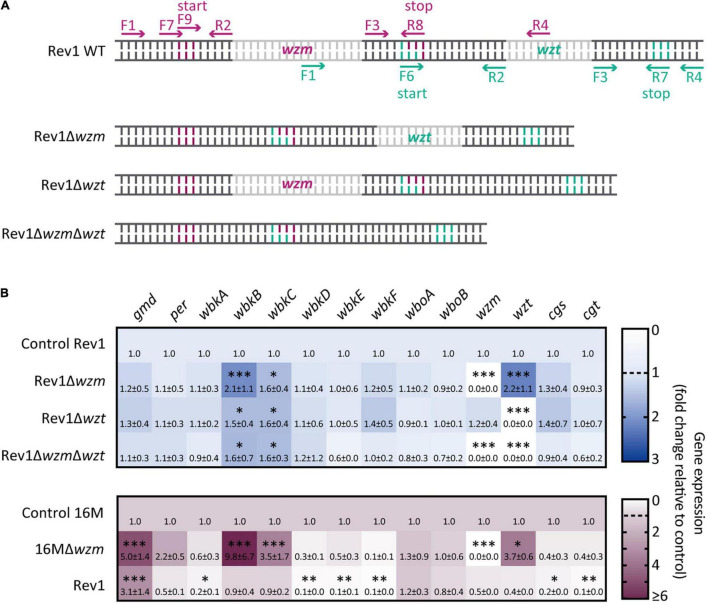
Deletion of *wzm* and/or *wzt* leads to different transcriptional regulations of O-PS biosynthesis genes. **(A)** Schematic representation of the forward (F) and reverse (R) primers designed onto *wzm* (pink) and *wzt* (green) to delete their inner regions (gray), with the respective start/stop codons in the wild-type (WT) parental strain and resulting mutants. **(B)** Heat maps representing the mean values of the genetic relative expression (fold-change; mean ± SD) determined by qRT-PCR in Rev1Δ*wzm*, Rev1Δ*wzt*, Rev1Δ*wzm*Δ*wzt* vs. Rev1 control (upper panel; *n* = 5), including 16MΔ*wzm* and Rev1 vs. 16M control (lower panel; *n* = 2) for comparative purpose. Fisher’s LSD test: ^***^p ≤ 0.001, ^**^p ≤ 0.01, *p ≤ 0.05 vs. Rev1 or 16M control (base line = 1).

After assessing the expected genotypes by sequencing for the Rev1Δ*wzm*, Rev1Δ*wzt*, and Rev1Δ*wzm*Δ*wzt* mutants (Figure 1A; Supplementary Figures 1, 2, Supplementary Table 3), we studied variations by qRT-PCR (Supplementary Table 4) for the relative expression of the genes *wzm*/*wzt*, *wbk*, and *wbo* O-PS biosynthesis clusters (Godfroid et al., 2000; González et al., 2008), as well as for *cgs* and *cgt*, which code for the synthesis and export to the periplasm of cyclic glucans. Of note, these sugars, together with the LPS, are envelope components involved in *Brucella* virulence (Haag et al., 2010; Guidolin et al., 2015). No transcription of *wzm* and/or*wzt* was detectable in the corresponding single- or double-deletion mutants (Figure 1B). As both genes are sequentially located in the chromosome (Godfroid et al., 2000), it is generally assumed that *wzm*/*wzt* mutations should cause an analogous effect and that the deletion of *wzm* would hinder *wzt* expression. Strikingly, however, Rev1Δ*wzm* showed significant (*p* < 0.001) overexpression of *wzt*, but Rev1Δ*wzt* showed unchanged expression of *wzm* (e.g., similar to the parental). In agreement with this, the *in silico* study revealed that the start codon of *wzt* overlaps the stop codon by one nucleotide. This overlap suggests the existence of a multiple reading frame, a feature conserved across microbial genomes and a common regulation mechanism (Johnson and Chisholm, 2004). Additionally, it has been proposed that these two genes could constitute a sole operon for other gram-negative bacteria (Rocchetta and Lam, 1997; Goldberg, 1999). Thus, it seems reasonable that a deletion upstream of *wzm* not only maintains but also alters the expression of *wzt*. In sum, we demonstrate that *wzm* deletion allows the transcription of *wzt* and causes its overexpression in Rev1Δ*wzm*.

Additionally, the three Rev1 *wzm*/*wzt* mutants showed enhanced expression (*p* < 0.05) of *wbkB* and *wbkC* with respect to Rev1 (note that *wbkB* encodes an enzyme of unconfirmed function, and, *wbkC*, a formyltransferase; Godfroid et al., 2000). This finding opens the possibility of antigenic changes in the nascent O-PS, although shortening would not be expected, as no signs of repressed expression were detected. Comparatively, 16M*wzm* showed not only overexpression of these three genes (e.g., *wbkB, wbkC, and wzt*) but also upregulation of *gmd* vs. the 16M parental strain, and this was not reproduced in Rev1*wzm*. With respect to the parental strains, Rev1 showed significant upregulation of *gmd* and downregulation of *wbkA*, *wbkD*, *wbkE*, *wbkF*, *cgs*, and *cgt* genes. Of note, *cgs* and *cgt* encode for cyclic glucans, which are involved in evading host immune response (Briones et al., 2001; Arellano-Reynoso et al., 2005; Haag et al., 2010; Roset et al., 2014), osmotic resistance (Roset et al., 2014), and oxidative and detergent pressure resistance (Mirabella et al., 2013). Thus, this result is consistent with the attenuation of Rev1.

### The Cytoplasmic O-Polysaccharide of Rev1 *wzm/wzt* Mutants Shows Antigenic Features

**FIGURE 2:**
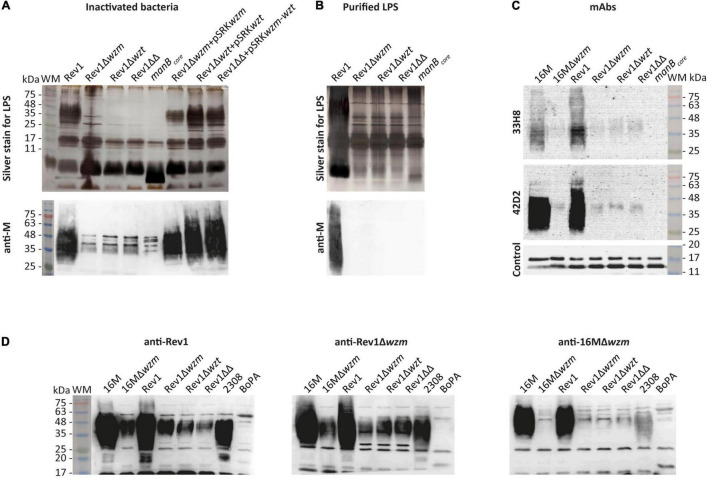
Rev1 *wzm*/*wzt* mutants express R-LPS with a complete core and a cytoplasmic O-PS reacting with mAbs. **(A)** Whole-cell phenol-inactivated bacteria or **(B)** purified LPS profiles detected by modified periodate-alkaline silver staining (upper panels) or by WB with anti-M monospecific serum (lower panels). **(C)** O-PS epitopes of whole bacteria recognized by the mAbs 33H8 for 20 min of exposure (the upper panel) or 42D2 for 6 min (the middle panel); anti-*B. ovis* serum was run as loading control on the same 42D2 blot (the lower panel). **(D)** Western blot with anti-Rev1, anti-Rev1Δ*wzm*, or anti-16MΔ*wzm* immune sheep sera. The images presented in the same groups were processed in parallel, and the results are representative of reproducible experiments. WM, pre-stained weight Marker VI (PanReac AppliChem, Castellar del Vallés, Barcelona, Spain).

In standard tests for *Brucella* typing (Alton et al., 1988), the three mutants showed the expected R-LPS phenotype, the typical small colonial size of Rev1 (Grilló et al., 2000) and Rev1 inhibition by penicillin G (P_5_) and safranin O (Saf_100_) (Supplementary Table 5). The complete core and the presence of O-PS in *wzm*/*wzt* mutants were evidenced by alkaline silver staining and Western blot with anti-M monospecific serum, respectively, in whole-cell inactivated bacteria (Figure 2A). We confirmed that the O-PS was not exposed at the mutant outer membrane (OM), since it was not detected using purified LPS samples (Figure 2B), as reported for other gram-negative bacteria (Guo et al., 2017; Moosavian et al., 2020). Using an anti-M monospecific serum for Western blotting (Figure 2A) revealed a faint, smeared band at 35–48 kDa in Rev1 mutants that were absent in BmH38R*manB*_*core*_ (control) and were very strong (25–75 kDa) in Rev1 and complemented strains. Further analysis with anti-C/Y 33H8 and 42D2mAbs showed O-PS in *wzm*/*wzt* mutants but at lower reactivity than in Rev1 and 16M (Figure 2C). As O-PS biosynthesis was not downregulated, differences in reactivity could be due to low amounts of O-PS in the mutants. This hypothesis is compatible with a capture of bactoprenol by the nascent non-translocated O-PS (Rocchetta and Lam, 1997), making it unavailable for further biosynthesis. Both *B. melitensis* S-LPS strains were less reactive with 33H8 than with 42D2 (which is evident even after extended exposure time), and Rev1 systematically exhibited stronger signals with 33H8 than with 16M (Figure 2C), evidencing different O-PS epitope compositions.

Rev1 mutants reacted strongly with sera from sheep infected with Rev1Δ*wzm*, showing a ≈35–48 kDa O-PS-smeared band as well as a distinct band at ≈29 kDa, which was also detected (faintly) by anti-Rev1 but not by anti-16MΔ*wzm* sheep sera (Figure 2D). This 29 kDa band was absent in 16M parental and mutant strains but observed in Rev1 by a Western blot with heat-treated anti-Rev1Δ*wzm* serum (data not shown). This result suggests the presence of an Omp that is unlinked in Rev1 and covalently bound to PG in 16M, as reported for *Brucella abortus* Omp2b and Omp25 (Godessart et al., 2021).

### Rev1 *wzm/wzt* Mutants Exhibit High Sensitivity to Antibiotics and Cationic Dyes

The presence of 10% CO_2_ inhibited the growth of 16MΔ*wzm*, as previously reported (Zabalza Baranguá, 2017), but not the growth of the Rev1 mutants. Also, the Rev1 mutants were more susceptible than Rev1 to streptomycin (Str_2_._5_), polymyxin B (PxB_1_._5_), and colistin (Col_4_), with no differences between mutants (Supplementary Table 6). Regarding the susceptibility to the antibiotics of choice for treating human brucellosis (Ariza et al., 1986), the three mutants were more susceptible than Rev1 to streptomycin and rifampicin in MIC and MBC_90_; furthermore, a synergistic effect with doxycycline was detected in a solid medium, particularly for streptomycin (*p* ≤ 0.001), leading to almost complete inhibition of mutant growth (Supplementary Table 7). These results suggest that, in case of accidental human inoculation, conventional treatment would be successful, in contrast to that reported for Rev1 (Blasco and Díaz, 1993; Grilló et al., 2006a).

**FIGURE 3:**
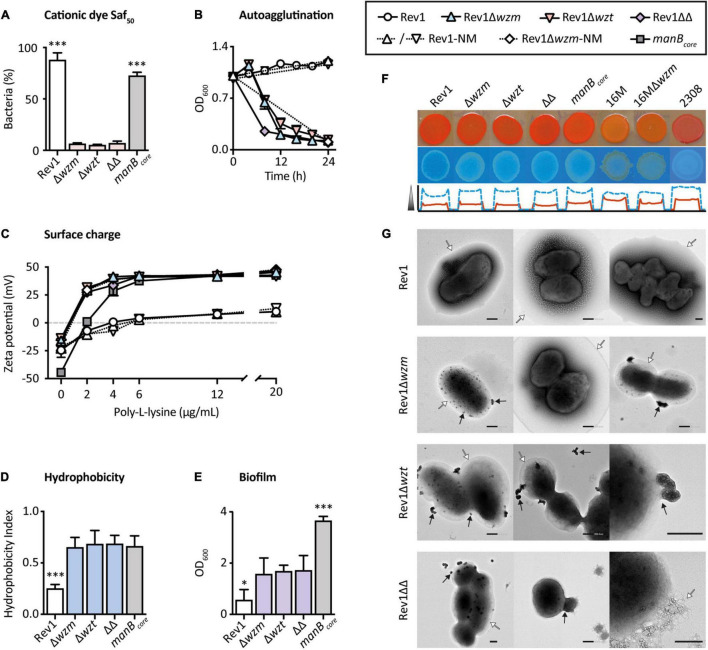
Outer membrane properties of *wzm*/*wzt* mutants are modified in comparison with Rev1. **(A)** Inhibition by the cationic dye safranin O at 50 μg/ml (Saf_50_) in BAB-S and 10% CO_2_ represented as surviving bacteria (%; mean ± SD; *n* = 3) vs. the standard growth condition. **(B)** Autoagglutination in static culture determined by spectrophotometry (mean ± SD; *n* = 2; representative result). **(C)** Surface charge measured by Zeta-potential (mean ± SD) in absence (*n* = 4) or presence (*n* = 2) of poly-L-lysine. **(D)** Hydrophobicity based on differential partitioning in aqueous-hydrocarbon solution (mean ± SD; *n* = 3). **(E)** Biofilm formation quantified by crystal violet staining (mean ± SD; *n* = 3). **(F)** EPS analysis by the intensity of Congo Red (upper row; red line) or Calcofluor (the low row; the discontinuous blue line). **(G)** Representative TEM images with negative stain in which EPS and OMVs are indicated by white and black arrows, respectively; scale bars = 200 nm. Fisher’s LSD test; ^***^p ≤ 0.001, *p ≤ 0.05 vs. the other groups.

The inhibition observed with Saf_100_, a basic monovalent cationic dye largely adsorbed by hydrophobic surfaces (Atun et al., 1998) as described for crystal violet-oxalate (Popescu and Doyle, 1996), could represent changes in the OM and cell walls (Jankowski et al., 2005). Thus, we quantified the susceptibility of the mutants to a lower concentration of this dye (Saf_50_), which gave only a 6% viability of mutants, in contrast to (*p* ≤ 0.001) the 87.8% of Rev1 parental strains (Figure 3A). Rev1 survival could be explained by the hydrophilic hindrance of the S-LPS (Nikaido and Vaara, 1985; Godfroid et al., 2000) and/or by buffering of the free radicals generated by this dye by CO_2_ (Jankowski et al., 2005). However, since BmH38R*manB*_*core*_ survived (72.5%) more than *wzm*/*wzt* mutants, safranin O susceptibility suggests that the OM undergoes a particular reorganization, resulting in more exposed lipid A and core moieties in the mutants (Vaara, 1992; Perevoshchikova et al., 2009; Clifton et al., 2016; Fontana et al., 2016).

### Blocking O-Polysaccharide Transport Causes Pleiotropic Changes Associated With the Outer Membrane and Cell Wall

Lipopolysaccharide biosynthesis is generally related to other cellular components involved in bacterial integrity (Morè et al., 2019). For instance, R-LPS and O-PS deficiencies have been associated with interrelated properties, such as spontaneous autoagglutination (Caro-Hernández et al., 2007), adhesion to solid layers (Nakao et al., 2012), increased hydrophobicity, and negative surface charge (González et al., 2008). Indeed, surface adhesion is dependent on the bacterial charge and the hydrophobic nature of the substrate, e.g., polystyrene (Fletcher and Loeb, 1979). Accordingly, these properties were found in the three mutants (Figures 3B–E).

Extracellular polymeric substances (EPS) are heterogeneous components typical of biofilm and stain differently by Congo Red or by Calcofluor (Wood, 1980). We found that both dyes bound moderately to Rev1 and its mutants, but only minimally to 16M and 16MΔ*wzm* (Figure 3F); these differences seem to be associated with the nature of the parental strain, as revealed by BmH38R*manB*_*core*_ and *B. abortus*2308 controls, and have been previously related to host preference and virulence (Uzureau et al., 2007). Although the acquisition of fine images of the native EPS is a major challenge due to its low contrast and tendency to collapse (Dohnalkova et al., 2011), we successfully detected the presence of EPS in Rev1 and its mutants by transmission electron microscopy (TEM), which revealed it to be a globular structure surrounding both fibrous and hydrated shapes (Figure 3G).

Other interesting structures promoting adhesiveness to solid surfaces in *Brucella* are the OM vesicles (OMVs), commonly called blebs (Godefroid et al., 2010). Blebs are nanovesicles released from the OM whose composition (Gamazo and Moriyón, 1987; Avila-Calderón et al., 2020; Ruiz-Palma et al., 2021) and amount (Solanki et al., 2021) have been associated with the lack of O-PS, and they are overproduced in strains that auto-agglutinate (Godefroid et al., 2010). As shown by TEM (Figure 3G), the mutants showed abundant OMVs as outward bulges protruding from the surface or as already detached vesicles, while the OM maintained its integrity. In stark contrast, we did not find even a single OMV in Rev1. These structural changes could indicate a rearrangement of cell envelope components.

**FIGURE 4:**
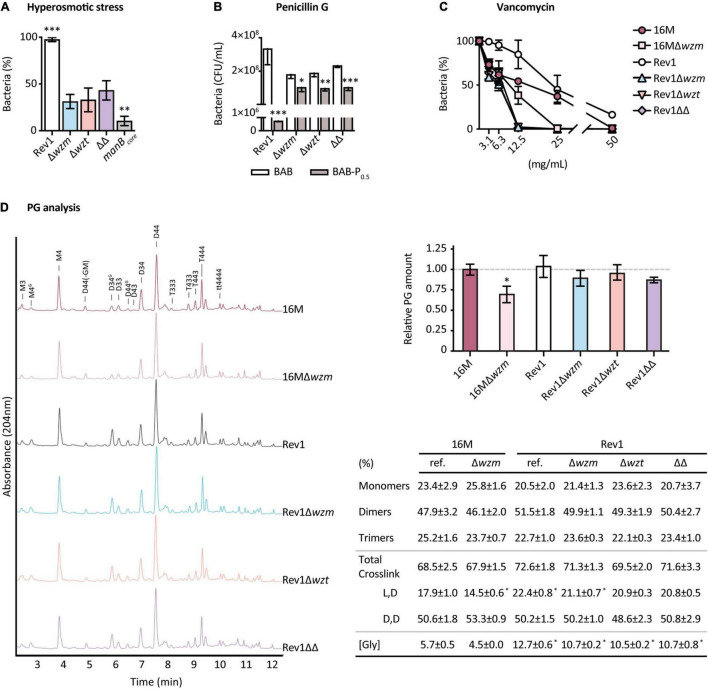
Rev1*wzm*/*wzt* mutants exhibit differences in cell-wall properties. Susceptibility to: **(A)** Hyperosmotic stress, expressed as the percentage of bacteria survival after incubation (37°C, 48 h) of 10^4^ CFU/ml in 0.5 M NaCl, plated in BAB; **(B)** Penicillin G expressed as the number of CFU/ml obtained after triplicate culturing (37°C, 8 days) of serial 10-fold dilutions from 10^9^ to 10^4^ CFU/ml in 0.5 IU/ml of penicillin G vs. BAB plates (P_0_._5_); and **(C)** Vancomycin, expressed as the percentage of bacteria survival after incubation (37°C, 1 h) of 10^4^ CFU/ml in twofold serial dilutions (50–3.125 mg/ml) of the antibiotic and triplicate plating in BAB; all results represent the mean ± SD (*n* = 3) of 2 or 3 independent experiments. **(D)** PG analysis as representative normalized chromatograms of three independent cultures of each strain, showing the relative PG amount and percentage of composition (mean ± SD; *n* = 3) of 16M and Rev1 reference and *wzm*/*wzt* mutant strains. Non-labeled peaks correspond to unknown muropeptides. Fisher’s LSD or *t*-tests; ^***^p ≤ 0.001, ^**^p ≤ 0.01, *p ≤ 0.05 vs. the other groups or vs. 16M in panel **D**.

The OM and the cell wall are covalently linked (Godessart et al., 2021), and their consistency is directly responsible for bacterial osmotic protection (Morè et al., 2019). In our studies, all strains grew well in sucrose hyperosmotic and hypoosmotic conditions; however, fewer than 50% of *wzm*/*wzt* mutants, and 10% of BmH38R*manB*_*core*_ survived in a hyperosmotic.5-M NaCl medium, while the growth of Rev1 was unaltered (Figure 4A), highlighting the importance of the selected osmolyte to be used (Cheung et al., 2009; Shabala et al., 2009; Pilizota and Shaevitz, 2013; Schuster et al., 2020). Even though EPS is involved in protecting against osmotic stress and antimicrobials (Dohnalkova et al., 2011), its presence did not mitigate NaCl stress in Rev1 *wzm*/*wzt* mutants.

Susceptibility to penicillin G can provide information about cell wall rearrangements, since this ß-lactam targets the penicillin-binding proteins (PBPs) involved in PG assembly (Kohanski et al., 2010), leading to the inhibition of PG transpeptidation and subsequent cell-wall destabilization (Wolter and Lister, 2013). We observed a total inhibition at P_5_ of 10^9^ CFU/ml for Rev1 as well as for its derivatives (Supplementary Table 5). Thus, we next sought to determine whether using lower concentrations could detect differences between Rev1 and its derivatives. All strains were significantly inhibited in a solid medium at P_0_._5_, but, surprisingly, the mutants were less inhibited than Rev1 (Figure 4B) and also showed higher MBC_90_ (0.84 vs. 42 IU/ml). As target modification is relatively unlikely, the increased resistance of mutants could be due: (i) to PBP being shielded—by its own substrate (Lepage et al., 1995) and/or by other cell products or structures (Livermore, 1987); or (ii) to reduced PBP activity, leading to PG remodeling (Peters et al., 2018; Morè et al., 2019). To further elucidate the PG structure, we studied the susceptibility of the mutants to vancomycin, a non-β-lactam glycopeptide that hinders PG assembly by blocking its precursors (Kohanski et al., 2010). Rev1 *wzm*/*wzt* mutants were more susceptible than the parental Rev1, showing total inhibition at 12.5 mg/ml (Figure 4C); this could indicate changes in the amount and/or composition of the PG that, in absence of the S-LPS building machinery in the periplasm, could facilitate vancomycin activity.

We therefore analyzed the PG of *B. melitensis* 16M and Rev1 and their derivatives by UPLC-MS (Figure 4D). For PG quantification, Rev1 *wzm*/*wzt* mutants presented lower amounts of PG (10–15% less) than the parental lines; reduction was even more remarkable (30%) for 16MΔ*wzm* vs. 16 M, in line with previous studies (Kreutzer et al., 1977). The fact that these mutants showed decreased PG confirms the hypothesis that truncating the Wzm/Wzt system impairs cell wall biogenesis, presumably due to sequestering the complex bactoprenol–O-PS when the transport is blocked. Furthermore, it would explain the higher susceptibility of the mutants to vancomycin. For the PG structure, we report for the first time the basic PG building block in *B. melitensis*, consisting of GlcNAc-MurNAc-L-Ala-D-Glu-mDAP-D-Ala-D-Ala, which is similar to that of other gram-negative bacteria (Vassen, 2018). *B. melitensis* PG included the presence of glycine (Gly) rather than Ala at Position 4 of the stem in a relatively small percentage of the muropeptides. For PG composition, we observed significant differences (*p* ≤ 0.05) between Rev1 and 16M parentals, as well as a general increase of L,D-crosslink and Gly in Rev1, suggesting a more active incorporation of glycine by L,D-transpeptidases (Ldts) than in 16M. Interestingly, 16MΔ*wzm* had a significant reduction (*p* ≤ 0.05) of the L,D-crosslink vs. 16M, a trend also observed for Rev1 *wzm*/*wzt* mutants vs. Rev1. These compensations underscore the essential, versatile, and functional coupling of LPS, OM, and cell wall biosynthesis, and strongly suggest that a block of O-PS transport causes pleiotropic effects on *Brucella*.

### Rev1 *wzm*/*wzt* Mutants Are Highly Attenuated in BALB/c Mice, Highlighting the Immunity Induced by Rev1Δ*wzm*

**FIGURE 5:**
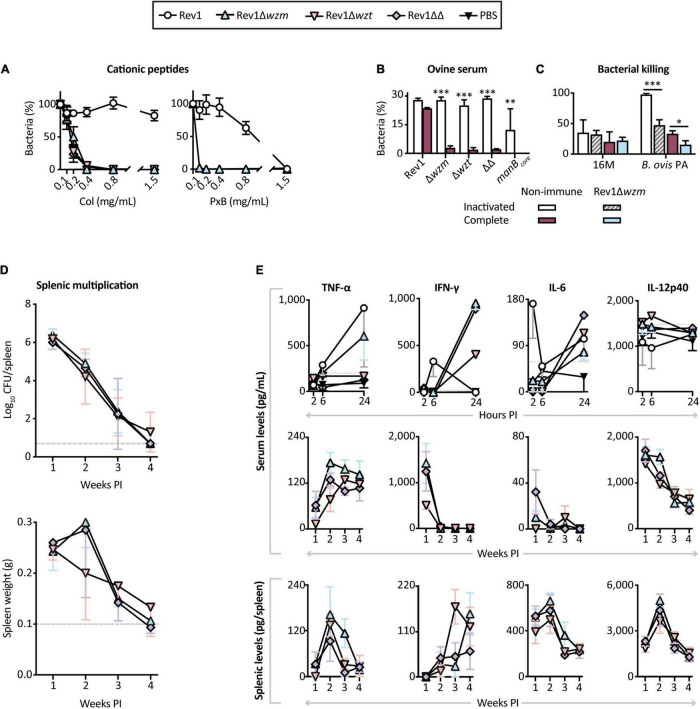
Rev1Δ*wzm* is highly attenuated and triggers a stronger adaptive immune response than Rev1Δ*wzt* or Rev1Δ*wzm*Δ*wzt* in the BALB/c mice. Susceptibility is shown for: **(A)** Polymyxin B (PxB) or colistin (Col), as models of cationic bactericidal peptides of the innate immune system (mean ± SD; *n* = 2); and **(B)** complement-mediated killing by conventional non-immune ovine serum, either complete or heat inactivated (mean ± SD; *n* = 2). **(C)** Bacterial killing activity of sera from sheep immunized with Rev1Δ*wzm* against *B. melitensis* 16M and *B. ovis* PA (BoPA) virulent strains (mean ± SD; *n* = 4). **(D)** Kinetics of spleen infections and weights (mean ± SD; *n* = 3 at 1 and 4 weeks PI, *n* = 4 at 2 and 3 weeks PI) of the BALB/c mice inoculated IP with 10^8^ CFU/mouse of the correspondent mutant; the dashed lines indicate the detection limit (log_10_ 5 CFU/spleen = 0.70) and normal splenic weight (0.1 g) in the BALB/c mice. **(E)** Cytokine profiles in blood sera and splenocytes supernatants from the same mice at selected intervals; sera collected at 2, 6, and 24 h PI (*n* = 5) were processed as pools, including two independent experiments for Rev1Δ*wzm*, Rev1, and PBS groups; dotted lines represent PBS’s maximum value; blood sera and splenocytes supernatants collected at 1, 2, 3, and 4 weeks PI were processed individually for each necropsied group (mean ± SEM). Fisher’s LSD test; ^***^p ≤ 0.001, ^**^p ≤ 0.01, *p ≤ 0.05 immune vs. non-immune sera for a given strain.

Resistance of virulent *Brucella* to soluble factors of the host immune system is essential for their survival *in vivo*. We therefore used *in vitro* models of susceptibility to polymyxins as antimicrobial cationic polypeptides, targeting the LPS phosphate groups (Martínez de Tejada et al., 1995), as well as to normal sheep serum as a source of complement. As previously reported (Stranahan and Arenas-Gamboa, 2021), R-LPS mutants were highly inhibited by PxB, Col, and serum complements (Figures 5A,B). Moreover, sera from Rev1Δ*wzm*-immunized sheep showed a marked killing effect on *Brucella ovis* rather than on *B. melitensis* (Figure 5C), a finding that could be explained by the natural absence of O-PS in *B. ovis* that makes their OM proteins more readily accessible to antibodies (Monreal et al., 2003).

In the BALB/c mice, the three mutants evidenced extreme attenuation. The vaccinated mice had high levels of spleen colonization at 1 week post-inoculation (PI) but were completely cleared of the mutants by 3–4 weeks (Figure 5D), in contrast to the longer persistence of Rev1 (Supplementary Figure 1). Furthermore, we reproducibly observed a peak of splenomegaly at 2 weeks PI in the mice vaccinated with Rev1Δ*wzm*, a less pronounced peak in the mice vaccinated with Rev1Δ*wzm*Δ*wzt*, and no peak in those vaccinated with Rev1Δ*wzt* (Supplementary Figure 1). This inflammatory response could be associated with an enhanced adaptive immune response mediated by cytokines, which are crucial in protective immunity against *Brucella* (Grilló et al., 2012; Sancho et al., 2014). We next analyzed cytokine production in both blood and spleen supernatants at early (≤24 h) and late (≤4 weeks) PI intervals (Figure 5E). Notably, the Rev1Δ*wzm* mice displayed high serum levels of circulating TNF-α and IFN-γ at 24 h PI, and of TNF-α and IL-12 at 2 weeks PI; likewise, the peak of splenomegaly at 2 weeks PI correlated with high levels of proinflammatory TNF-α, IL-6, and IL-12 in these spleens, and a peak of IFN-γ at 4 weeks PI, which was delayed with respect to that described for Rev1 (Sancho et al., 2014). This cytokine relation triggered by Rev1Δ*wzm* plays a critical role in limiting intracellular replication and quick clearance of the mutant as well as in triggering a protective Th1 immune response (Baldwin and Goenka, 2006; Dorneles et al., 2015; Jain-Gupta et al., 2019). Thus, we demonstrate that Rev1Δ*wzm* does not require long-lasting persistence to induce protective immunity, in contrast to what is generally accepted for *Brucella*, where Rev1 immunogenicity is dependent on its persistence in mice spleens (Bosseray and Plommet, 1990; Grilló et al., 2000; Moriyón et al., 2004; Barrio et al., 2009; OIE, 2018).

### Rev1Δ*wzm* Is as Effective as Rev1 Against *Brucella melitensis* and *Brucella ovis* in BALB/c Mice

On the basis of its immunogenicity, we studied whether the splenomegaly induced by Rev1Δ*wzm* and the subsequent adaptive immune responses were dose dependent (Supplementary Figure 1). Immunization with 10^6^ to 10^8^ CFU/mouse of Rev1Δ*wzm* induced equivalent spleen infections, but only 10^8^ CFU achieved a homogeneous intragroup infection and splenomegaly, similar to that induced by Rev1 in standard conditions. This confirmed that the immune response to Rev1Δ*wzm* was dose dependent.

**FIGURE 6:**
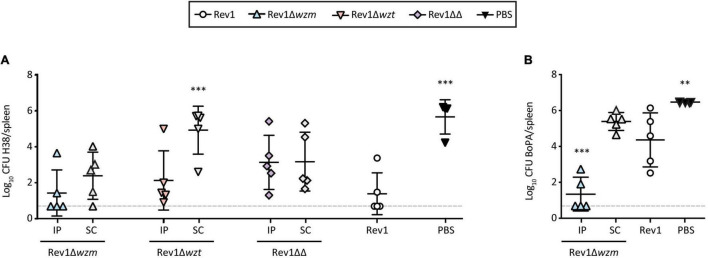
Rev1Δ*wzm* protects against virulent *B. melitensis* and *B. ovis* challenges in the BALB/c mice. Efficacy in the BALB/c mice (*n* = 5) IP or SC vaccinated with Rev1 *wzm*/*wzt* mutants (10^8^ CFU/animal) or with Rev1 reference vaccine (10^5^ CFU/animal, SC) or PBS as controls; after 4 weeks, the mice were IP challenged with **(A)** 1 × 10^4^ of *B. melitensis* H38:Gm^r^ (H38) or **(B)** 5 × 10^5^ of *B. ovis* PA:Gm^r^ (BoPA:Gm^r^). After 2 or 3 weeks, respectively, the mean ± SD (*n* = 5) log_10_ CFU of the challenge strain in spleens was determined. Detection limit (log_10_ 5 CFU/spleen = 0.70) is indicated with a discontinuous line. Fisher’s LSD test; ^***^p ≤ 0.001, ^**^p ≤ 0.01 vs. Rev1 control.

Efficacy studies on mice use intraperitoneal (IP) or subcutaneous (SC) vaccinations as screening or more exigent models for R-*Brucella* strains, using Rev1 in standard conditions and PBS as efficacy and non-vaccinated controls, respectively (González et al., 2008). Accordingly, we used both routes to evaluate Rev1 *wzm*/*wzt* mutants in the BALB/c mice. Against a *B. melitensis* H38 (S-LPS) challenge, Rev1Δ*wzm* was as protective as Rev1, while Rev1Δ*wzt* failed by the SC route and Rev1Δ*wzm*Δ*wzt* induced heterogeneous responses (Figure 6A). Against a *B. ovis* PA (R-LPS) challenge, Rev1Δ*wzm* administered *via* SC was also as effective as Rev1, and IP improved the protection conferred by the standard Rev1 vaccine control (Figure 6B).

Differences in the immunogenic properties of Rev1 *wzm*/*wzt* mutants could be attributable to a differential presence of Wzm or Wzt, supporting the hypothesis that Wzt has a crucial role in conferring immunity (Wang et al., 2014b). Indeed, we detected *wzt* overexpressed in Rev1Δ*wzm*. Although the overlap of the start/stop codons could lead to a coupled translation of both proteins, an increase at the translational level could be relevant to O-PS antigenicity. Since Wzt is incorporated into the cytoplasm when Wzm is missing (Singh et al., 2013; Mohammad et al., 2016), the accumulation of nascent O-PS molecules in Rev1Δ*wzm*, presumably attached to the cytoplasmic membrane by the lipid carrier, might have positive feedback on Wzt molecules. Additionally, as Wzt is responsible for O-PS terminal recognition (Valvano, 2003, 2015; Clarke et al., 2004; Cuthbertson et al., 2005, 2007; Hagelueken et al., 2015; Williams et al., 2017; Bi and Zimmer, 2020), its binding to the O-PS could be necessary to provide the antigen with its final conformation and thus with its immunogenic properties.

### Rev1Δ*wzm* Has a Similar Growth Pattern as Rev1 and Is Stable After *in vitro* and *in vivo* Subculturing

**FIGURE 7:**
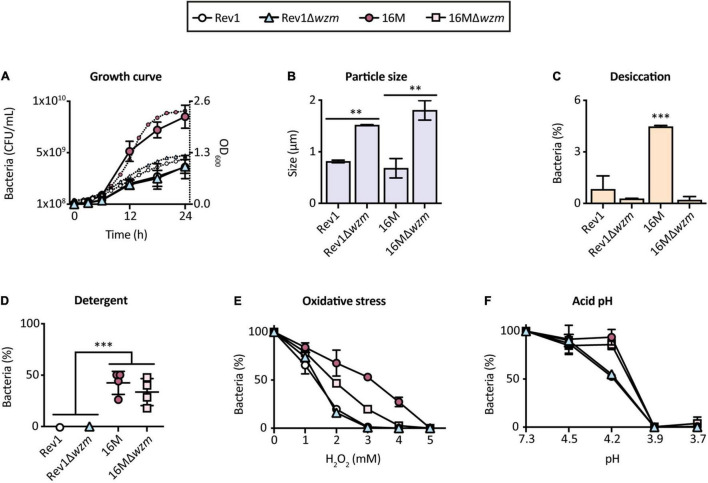
Rev1Δ*wzm* has larger particle size than Rev1, but both perform similarly in the *in vitro* growth curves and environmental susceptibility. **(A)** Bacterial growth curves determined by the number of viable cells (CFU/ml; the left axe, large symbols) and medium absorbance (OD_600_; the right axe, small symbols). **(B)** Particle size values measured by Zetasizer (μm). Percentage of bacterial inhibition by: **(C)** Desiccation, **(D)** Detergents (0.1% Triton X-100), **(E)** Oxidative (H_2_O_2_), and **(F)** Acidic pH pressures, as models of stress encountered by *Brucella* in the natural environment and in the host niche. All results are expressed as the mean ± SD of at least three measures; strains Rev1, 16M, and 16MΔ*wzm* were included as controls in all the experiments; the percentages of surviving bacteria (%) were determined with respect to the initial suspension. Fisher’s LSD test; ^***^p ≤ 0.001, ^**^p ≤ 0.01 vs. the indicated groups.

Having selected Rev1Δ*wzm* as a promising vaccine candidate, we sought possible *in vitro* defects that could affect the bacterial viability and/or scale-up production properties. We determined that Rev1Δ*wzm* resembled the growth curve of Rev1 yet provided higher turbidity and larger particle size (Figures 7A,B), probably due to the presence of OMVs protruding from the mutant, as evidenced by TEM (Figure 3G). Additionally, Rev1Δ*wzm* had a similar susceptibility as Rev1 to desiccation, detergents, oxidative stress, and acidic environment (Figures 7C–F), as well as to lyophilization. These environmental susceptibilities of Rev1 (which have not previously been reported) and its mutant agreed with the detected downregulation of cyclic glucan genes as compared to the virulent strain 16M (Figure 1B). Furthermore, we detected unaltered genotypical [*wzm* deletion and GI-2/*wbk* regions integrity (Mancilla et al., 2013)] and phenotypical (including inner O-PS production) features after 20 *in vitro* passages as well as after 5 consecutive passages in mice spleens. In contrast, Rev1 viability was significantly reduced after 15 *in vitro* passages, supporting the recommendation of minimizing *in vitro* propagation for quality control (Grilló et al., 2012). These described results (data not shown) suggested that Rev1Δ*wzm* is not likely to have antigenic drift.

### Rev1Δ*wzm* Is Attenuated in BeWo Cells and Safe in Pregnant Mice

**FIGURE 8:**
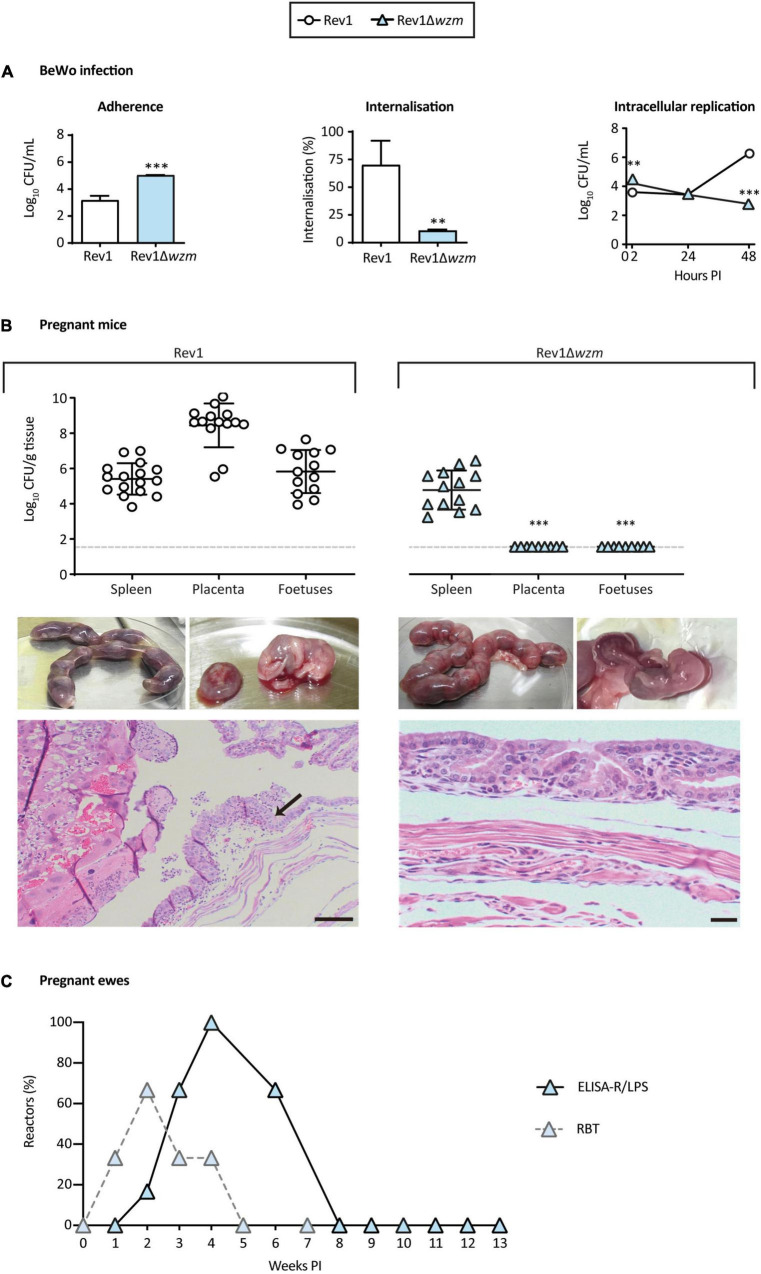
Rev1Δ*wzm* is attenuated in BeWo cells, safe in pregnant mice, and induces minimal serological interference in ewes vaccinated at mid-pregnancy. **(A)** Adherence, internalization, and intracellular multiplication in BeWo cells (*n* = 3). **(B)** Bacteriology of spleens (upper panel), as well as macroscopic and microscopic representative images of placentas and fetuses from the pregnant CD1 mice, analyzed at 14 days after IP inoculation with 1 × 10^7^ CFU of Rev1Δ*wzm* (*n* = 14; the right column) or 1 × 10^6^ CFU of Rev1 (*n* = 16; the left column). The arrow indicates leukocyte infiltration; scale bars = 100 μm. Detection limit (log_10_ 33 CFU/g tissue = 1.52) is indicated with a discontinuous line. In panels **A,B**, results are presented as mean ± SD of the correspondent n value. **(C)** Serological response (% reactors) in ewes vaccinated SC with 1.8 × 10^10^ CFU of Rev1Δ*wzm* at mid-pregnancy, developing antibodies in ELISA-R/LPS and RBT. Fisher’s LSD or *t*-tests; ^***^p ≤ 0.001, ^**^p ≤ 0.01 vs. Rev1 control.

*Brucella* infection, including Rev1 (Jiménez de Bagüés et al., 1989), targets the reproductive tract, starting in trophoblast giant cells, disseminating to placenta and fetuses and ending in spontaneous abortion. As preclinical models, we studied the ability of Rev1Δ*wzm* to infect BeWo cells (a trophoblast-derived, choriocarcinoma cell line) and mouse placentas/fetuses as compared to the ability of Rev1. In BeWo cells, Rev1Δ*wzm* was more adherent but was internalized less efficiently after adherence and replicated less than Rev1 (Figure 8A). In turn, Rev1Δ*wzm* was not found in mouse placentas/fetuses, even after inoculation at a 10-fold higher dose than Rev1, although both strains resulted in similar spleen infections (Figure 8B). Histologically, Rev1Δ*wzm* enabled normal placentas with minimal neutrophilic infiltration and normal fetus viability, differing from Rev1 by its marked macroscopic edema, destruction of placental epithelium, and leukocyte infiltration in yolk sac. This mouse model has been successfully used as screening of pathogenicity (Grilló et al., 2012; Poveda-Urkixo et al., 2022), indicating that Rev1Δ*wzm* might be safer than Rev1 in pregnant animals and justifying further experiments in the natural host.

### Rev1Δ*wzm* Is Safe in Pregnant Ewes and Their Offsprings, and Induces Minimal Serological Interference

All ewes vaccinated with a high dose of Rev1Δ*wzm* at mid-pregnancy (*n* = 6) showed normal clinical parameters, mild and transient local reactions at the inoculation site, with no shedding the mutant through vagina or milk, during pregnancy or lactation, and had normal parturitions at 144 ± 3.3 days of pregnancy. Likewise, at necropsy, the mutant was not found in any ewe or offspring. These results contrasted with those reported for Rev1 (i.e., shedding by vagina during pregnancy, bacteria in placentas at parturition and in milk, placental necrosis, abortions, and vertical transmission to lambs) (Jiménez de Bagüés et al., 1989; Blasco, 1997; Hensel et al., 2020).

Serologically, all ewes reacted in ELISA-R/LPS after vaccination and turned negative before 8 weeks PI; a 66.7% also reacted in RBT (16.7% CFT positive), a percentage that decreased progressively until turning all ewes negative before 5 weeks PI (Figure 8C). These reactions were significantly less interferent than those reported for Rev1 (Barrio et al., 2009).

### The Impact of the Rev1 Background on Rev1Δ*wzm* Vaccine Properties Was Unpredictable

It is commonly assumed that vaccine candidates should retain some residual virulence to be efficient (Grilló et al., 2000). Indeed, just those 16M *wzm* mutants that show an attenuation pattern similar to Rev1 are as protective as Rev1 against a *B. melitensis* virulent challenge (Zabalza Baranguá, 2017), while 16MΔ*wzt* is less persistent and less protective than Rev1 (Wang Z. et al., 2014). On this basis, the high attenuation of Rev1Δ*wzm* should have led to failed efficacy; however, and, unpredictably, Rev1Δ*wzm* (but not Rev1Δ*wzt*) triggered a splenomegaly associated with a particular Th1 cytokine balance, as well as to a protective efficacy against virulent challenges. Besides the genetic and phenotypic divergences described between the Rev1 and 16M parentals related to virulence attenuation (Issa and Ashhab, 2016; Salmon-Divon et al., 2018, 2019; Kornspan et al., 2020), we have now described additional differences in genetic transcription, antigenicity evidenced by Western blot, and susceptibility to antibiotics and environmental stress factors. Indeed, when *wzm*/*wzt* mutations were applied to other brucellae (Wang et al., 2014a; Grilló et al., 2017; Aragón-Aranda et al., 2020; Lalsiamthara et al., 2020), neither mutant was as immunogenic as Rev1Δ*wzm*. All these findings demonstrate the relevance of the background in vaccine properties, as well as the unpredictable efficacy results obtained with Rev1Δ*wzm*.

## Conclusion

The Wzm/Wzt system is broadly conserved among gram-negative bacteria, and its inactivation in Rev1 not only restrains the export, quantity, and availability of O-PS but also triggers phenotypic changes of the OM and cell wall. Despite similarities *in vitro*, deletion of Wzm or Wzt transporter moieties elicited different immune responses and efficacies against *Brucella*-virulent infections. Indeed, Rev1Δ*wzm* displays superior vaccine properties: attenuation, immunogenicity, efficacy against a virulent infection in mice, safety in pregnant mice, high susceptibility to diverse stresses and antimicrobials, and safety and minimal serological interference in pregnant ewes. These results (summarized in Supplementary Figure 2) thus highlight a new concept that is essential in vaccine development, i.e., that a low persistence is not at odds with efficacy.

## Materials and Methods

### Strains and Culture Conditions

Bacterial strains (Supplementary Table 1) were stored at –20°C in 10% skimmed milk with 3% lactose (PanReac AppliChem, Castellar del Vallés, Barcelona, Spain) and routinely cultured at 37°C in normal atmosphere (air), using Trypticase Soy Broth (TSB; Condalab) at 150 rpm, or in plates of TSB supplemented with 1.5% bacteriological agar (TSA; Pronadisa) for *Escherichia coli* strains, or Blood Agar Base No. 2 (BAB; Oxoid) for *Brucella* spp., either plain or supplemented with 5% newborn calf serum (S; Gibco), 5% sucrose (Suc_5_; VWR Chemicals, Radnor, PA, United States), and/or antibiotics (Sigma-Aldrich, San Luis, MO, United States), such as kanamycin (50 μg/ml; Km_50_), polymyxin B (1.5 μg/ml; PxB_1_._5_), colistin (4 μg/ml; Col_4_), gentamycin (15 μg/ml; Gm_15_), streptomycin (2.5 μg/ml; Str_2_._5_), doxycycline (0.02 μg/ml; Dx_0_._02_), rifampicin (0.4 μg/ml; Rf_0_._4_), and penicillin G at (5 IU/ml; P_5_) or (0.5 IU/ml; P_0_._5_), as needed. Suspensions were adjusted by spectrophotometry (SmartSpec Plus; Bio-Rad, Hercules, CA, United States) in sterile TSB or PBS (pH, 7.2; VWR Chemicals, Radnor, PA, United States), as described elsewhere (González et al., 2008). The exact number of viable counts was determined retrospectively by serial dilutions in PBS and plating (0.1 ml, done in triplicate).

### Sequence Analysis and DNA Manipulation

*In silico* studies were performed with BLAST (Altschul et al., 1990), NCBI (Sayers et al., 2022), and KEGG (Kanehisa and Goto, 2000) databases. PCR primers (Supplementary Table 2) were synthesized by Sigma-Aldrich Química SL (Madrid, Spain). Vector sequences were obtained from Addgene (Kamens, 2015). Vector or chromosomal DNA purifications were performed by *miniprep* with E.Z.N.A. Plasmid Mini Kit I (Omega Bio-tek, Norcross, Georgia) or DNeasyUltraClean Microbial Kit (Qiagen, Düsseldorf, Germany). Single-colony DNA was extracted by boiling and centrifugation. PCR products were purified using the ATP Gel/PCR DNA Fragment Extraction Kit (ATP Biotech Inc., Taipei, China). DNA was quantified with a NanoDrop Spectrophotometer (Thermo Fisher Scientific, Waltham, MA, United States) and sequenced by STAB VIDA (Caparica, Portugal).

### Construction and Complementation of Single and Double *wzm/wzt* Rev1 Mutants

In-frame deletion mutants were obtained as previously described (Conde-Álvarez et al., 2012), using the suicide plasmids pJQKmΔ*wzm* (Zabalza Baranguá, 2017) and/or pJQKmΔ*wzt*. The latter was constructed using 16M genomic DNA and the designed primers (Supplementary Table 2) to amplify 198 bp upstream and *wzt* 1-66 codons with F1-R2, and *wzt* 209–253 codons and 193 bp downstream with F3-R4 *wzt*. These fragments were fused by overlap PCR with F1-R4 *wzt*, cloned in pCR2.1 (TOPO^®^TA Cloning, ThermoFisher Scientific) and sequenced. After using pCR2.1Δ*wzt* to transform *E. coli* TOP10F’, Δ*wzt* was subcloned into pJQKm (Quandt and Hynes, 1993; Scupham and Triplett, 1997) *Bam*HI and *Xba*I sites. Clones were screened by PCR (Supplementary Table 2), and representative non-mutated (NM) ones from each mutagenesis were kept as controls. For complementation, transconjugants, including the pSRK vector with *wzm* (Zabalza Baranguá, 2017), *wzt* or *wzm-wzt* (subcloning of BMEI1415 or BMEI1415-BMEI1416 into *Spe*I and *Xho*I sites) were selected in Km_50_ plates and, for pSRK*wzm-wzt*, 0.1-mM IPTG (Invitrogen, Waltham, MA, United States), and checked by PCR.

### Transcriptional Analysis

Genetic expression was analyzed from five independent extractions of Rev1 and derivatives, or 2 of 16M and 16MΔ*wzm* for comparative purposes. Total RNA from exponential cultures was extracted by lysozyme (Sigma-Aldrich, San Luis, MO, United States), proteinase K (Merck, Darmstadt, Germany), Zwittergent (Merck, Darmstadt, Germany), RNeasy Mini kit (Qiagen, Düsseldorf, Germany), and DNase [Ambion, Austin, TX, United states (ThermoFisher)] treatments, and its quality checked with Gel Loading Buffer II [Ambion, Austin, TX, United states (ThermoFisher)], in an agarose gel by confirming that the intensity ratio of 23S:16S proteins was 2:1, obtaining the cDNA with PrimeScript RT kit (Takara, Saint-Germain-en-Laye, France). The qRT-PCR was performed with SYBR Premix Ex Taq (Takara, Saint-Germain-en-Laye, France) in duplicate in an AriaMx real-time PCR system (Agilent) using 96-well microplates (Axygen, New York, NY, United States) as follows: 10 min at 95°C, 45 cycles of 15 s at 95°C, and 1 min at 60°C. Primers (Supplementary Table 4) efficiency (0.9–1.1) was assessed as 10^(–1/slope*ofCtvs*.*DNAdilutions*)^–1. Data were analyzed with Agilent AriaMx v. 1.2 software (Santa Clara, CA, United States) and relative transcription normalized by the 2^–ΔΔCt^ method using *IF-1* gene and representing the fold-change over Rev1 or 16M controls.

### Characterization of Rev1 *wzm/wzt* Mutants

Conventional *Brucella* biotyping was performed following the standard protocols (Alton et al., 1988). Briefly, the mutants were submitted to tests of catalase, oxidase, urease, agglutination with acriflavine, agglutination with anti-A/anti-M/anti-R monospecific sera, lysis by bacteriophages Tb, Wb, Iz, and R/C, susceptibility to thionine, fuchsine, and safranin O dyes. Also, we analyzed purity and homogeneity of colony size and the LPS phase by the crystal violet-oxalate test (Grilló et al., 2000). The *in vitro* growth curves were assessed by duplicate as turbidity and CFU/ml from suspensions adjusted to OD_600_ = 0.1 (TSB, 37°C, 150 rpm) at the selected intervals.

MIC/MBC_90_ to the selected antibiotics were determined in the cation-adjusted Müller–Hinton medium (M-H; BD) by the standard broth microdilutions method (EUCAST: European Committee for Antimicrobial Susceptibility Testing, 2003), using Rev1 and *E. coli* K12 as controls. Susceptibility to Dx_0_._02_, Gm_0_._125_, Rf_0_._4_, Str_2_._5_, PxB_1_._5_, Col_4_, P_0_._5_, and Saf_50_ was quantified by seeding suspensions between 10^9^ and 10^4^ CFU/ml by triplicate in BAB or BAB-S plates incubated (37°C, 8 days) under normal air or 10% CO_2_ atmosphere. Results were expressed as the% of bacterial survival vs. the standard culture (BAB, air). Resistance to vancomycin (Sigma-Aldrich, San Luis, MO, United States) was determined by incubating a suspension of 10^4^ CFU/ml in PBS with twofold serial dilutions (50–3.125 mg/ml) of the antibiotic (37°C, 1 h, 100 μL each, by duplicate) and plating in BAB to determine the% of surviving bacteria vs. control.

Analyses of the R-LPS and internal O-PS were performed with ≈10^10^ CFU/ml of whole bacteria inactivated by 0.5% phenol (72 h, 37°C, 150 rpm) or purified LPS by an extraction kit (Intron Biotechnology, Seongnam, South Korea’s) in SDS-PAGE and further modified periodate-alkaline silver staining or WB (Tsai and Frasch, 1982; Monreal et al., 2003). The latter was revealed with primary antibodies: (i) mAbs (1:2,000) anti-C/Y 33H8 or 42D2 (batch No. 051119 or 300502, Ingenasa), (ii) anti-M monospecific polyclonal rabbit serum (1:100; IdAB collection), or (iii) anti- *B. ovis*, Rev1, Rev1Δ*wzm* (either normal or heat treated 1 h, 56°C) or 16MΔ*wzm* sera from sheep experimentally infected (1:400; IdAB collection); and secondary HRP-conjugated antibodies: (i) an anti-mouse at 1:3,000 (Bio-Rad, Hercules, CA, United States cat. No. 170-6516), (ii) an anti-rabbit at 1:2,500 (Bio-Rad, Hercules, CA, United States cat. No. 170-6515), or (iii) protein G at 1:1,000 (Pierce cat. No. 21193), all diluted in 1% milk PBST. The images were acquired with ECL Kit (Bio-Rad, Hercules, CA, United States) in a ChemiDocSyngene with GeneSnap 7 (Frederick, MD, United States) or Bio-Rad with Quantity One (Hercules, CA, United States) software. Brightness and contrast adjustments were applied uniformly using GNU Image Manipulation Program v. 2.10 (open source^2^).

To assess the lack of spontaneous mechanisms of dissociation described for Rev1 (Mancilla et al., 2013), the integrity of GI-2 and *wbk* regions was determined after serial subcultures in BAB under normal or 10% CO_2_ atmospheres by conventional PCR with the primers presented in Supplementary Table 2; the amplified products were 353 bp (P1–P3) and 1,016 bp (P5–P7) in case of gene integrity, or 586 bp (P1–P2) and ≈1,400 bp (P5–P6) in case of excision.

### Study of Envelope-Related Properties

Autoagglutination was assessed by OD_600_ readings as described (Caro-Hernández et al., 2007) in at least two independent experiments. Bacterial particle size and surface charge of phenol-inactivated bacteria (37°C, 150 rpm, 24 h) adjusted to OD_600_ = 0.2 (7,000 × *g*, 4°C, 15 min) were measured in CsCl/HEPES as elsewhere (González et al., 2008) in absence or presence of poly-L-lysine (P6516; Sigma-Aldrich, San Luis, MO, United States) in a Zetasizer Ultra (Malvern Panalytical, Malvern, United Kingdom) at 25°C, using the ZS-XPLORER v. 2.01 software (United Kingdom).

Hydrophobicity was determined by measuring bacterial adherence, using a hydrocarbons method (Rosenberg et al., 1980), modified to avoid cell degradation due to xylene toxicity (Czerwonka et al., 2016). Phenol-inactivated cultures were centrifuged (6,000 × *g*, 4°C, 10 min) and washed with a PUM buffer; then, 2 mL adjusted to OD_470_ = 1 was mixed with an equal volume of xylene (PanReac AppliChem, Castellar del Vallés, Barcelona, Spain) by triplicate. After a vortex (5 s)-incubation (37°C, 10 min)-vortex (30 s)-incubation (room temperature; RT, 2 h) step, the absorbance of the low phase was measured to determine the hydrophobicity index as: 1 – (OD_470_/1).

Biofilm formation was studied by bacterial adhesion to polystyrene, cellular viability, and Calcofluor and Congo Red assays. Briefly, 200 μL with 5 × 10^8^ CFU/ml in TSB was incubated (37°C, 3 weeks) in 96-well plates (Sarstedt) by triplicate and, after PBS washings, was stained with crystal violet (PanReac AppliChem, Castellar del Vallés, Barcelona, Spain; 0.05% in water, RT, 15 min). The stain was washed with distilled water and dissolved in ethanol (Merck, Darmstadt, Germany) to measure adhesion as OD_600_. The viability of the attached bacteria was assessed by swabbing and culturing in BAB. EPS production was evaluated by seeding 20 μL of 10^9^ CFU/ml in BAB with 0.1% Calcofluor White M2R (Sigma-Aldrich, San Luis, MO, United States) or 4% Congo Red (Merck, Darmstadt, Germany); after 3 weeks, at 37°C, the intensity of Calcofluor’s fluorescence measured in a UV transilluminator (UVP) and color shift to intense red/black were visualized and quantified with ImageJ/Fiji v. 2.1 software (Schindelin et al., 2012).

The osmotic resistance was studied by incubating (37°C, 48 h) 10^4^ CFU/ml in TSB: water (1:5; hypoosmotic) or in 0.5 M NaCl (Dong et al., 2015) or 0.34-M Suc (Roset et al., 2006) (VWR Chemicals, Radnor, PA, United States; hyperosmotic) by triplicate. The CFU/ml was determined in BAB to calculate the % of bacterial survival vs. the initial suspension in two independent experiments.

### Susceptibility to Environmental Factors

Tolerance to desiccation was studied by keeping (RT, 6 days) a suspension (200 μL/well, 10^9^CFU/ml, TSB) completely dried by evaporation into 12-well plates (Sarstedt) by triplicate and rehydrating the pellet in PBS to determine the viable CFU/ml in BAB. Susceptibility to detergents was firstly evaluated with SDS (Merck, Darmstadt, Germany), since this surfactant inactivated all the *Brucella* tested at 0.06%; bacterial suspensions (10^4^CFU/ml, 100 μL, by triplicate) were cultured in BAB with 0.1% Triton X-100 (Sigma-Aldrich, San Luis, MO, United States). Oxidative stress and acid pH resistances were analyzed by incubating (37°C, 1 h) a 10^4^ CFU/ml suspension in TSB with an equal volume (100 μL) of H_2_O_2_ at increasing concentrations up to 5 mM or with acidified TSB at a final pH from 7.3 to 2.3 by duplicate. Results were expressed as bacteria (%) vs. control.

### Transmission Electron Microscopy

Massive *Brucella* cultures (37°C, 24 h) were harvested, washed (7,000 × *g*, 10 min) two times with sterile Sorenson’s phosphate buffer pH 7.4 (Kuo, 2007) and fixed (4°C, overnight) with 2.5% glutaraldehyde (Grade I, 70%; Sigma-Aldrich San, Luis, MO, United States) and 2% paraformaldehyde (powder, Sigma-Aldrich, San Luis, MO, United States) in a buffer. Samples were adsorbed (1:200 dilution, 3 μL, 1 min) in hydrophilized carbon-coated copper grids (glow discharge 5 mA, 20 s, Leica EM ACE200). After negative staining (1% uranyl acetate, 10 s, two times), representative images were acquired (JEOL JEM 1400 Plus).

### Ultra-Performance Liquid Chromatography-Mass Spectrometry

Peptidoglycan was purified from three biological replicas normalized to OD_600_ = 1 and analyzed, using 16M as control, as described elsewhere (Desmarais et al., 2013; Alvarez et al., 2016, 2020) on a Waters UPLC system (Waters Corporation, Milford, MA, United States) equipped with an ACQUITY UPLC BEH C18 Column, 130 Å, 1.7 μm, 2.1 mm × 150 mm (Waters, United States) and a dual wavelength absorbance detector. Muropeptide identity was confirmed by MS/MS analysis, using Xevo G2-XS QT of system (Waters Corporation, Milford, MA, United States). Quantification of muropeptides was based on their relative abundances normalized to the total amount of PG. Unidentified muropeptides and minor peaks with a relative abundance lower than 0.5% were excluded from further analysis.

### Susceptibility to Soluble Factors of the Immune System

Susceptibility to polymyxins B and E (colistin) as a model of cationic peptides and to normal ovine serum (IdAB collection) as a source of complement after 18 h of incubation was determined by duplicate as reported (Martínez de Tejada et al., 1995; González et al., 2008). Likewise, the *in vitro* bacterial killing properties of serum from sheep immunized with Rev1Δ*wzm* (IdAB collection) were studied against *B. melitensis* 16M (S-LPS) or *B. ovis* PA (R-LPS) virulent strains; normal sheep serum and PBS or PBS-S for *B. ovis* PA were used as controls to determine survival (%) in two independent experiments.

### BeWo Cells Studies

Bacterial adhesion, internalization, and intracellular multiplication in the BeWo (ATCC CCL-98; Sigma-Aldrich, San Luis, MO, United States) trophoblast-like cell line were determined as described previously (Castañeda-Roldán et al., 2004; Salcedo et al., 2013; Poveda-Urkixo et al., 2022). Briefly, 5 × 10^4^ cells/well were cultured in 12-well microtiter plates (Sarstedt) with an enriched F-12K medium (Kaighn’s Modification) supplemented with 10% of inactivated FBS and 2-mM L-glutamine (Glutamax 100×) (Gibco). The cells were infected at an MOI of 100, and, after 30 min of incubation, the adhered bacteria were killed with Gm_50_ (1.5-h exposure). After cells lysis with 0.1% Triton X-100 (Sigma-Aldrich, San Luis, MO, United States), the number of CFU/ml was determined in both treated (intracellular) and untreated (total) wells by plating in BAB to calculate (i) adhesion as log_10_ CFU/ml = total CFU-intracellular CFU; (ii)% of internalization = (intracellular CFU/total CFU) × 100; and (iii) intracellular multiplication as log_10_ CFU/ml found at 2, 24, and 48 h.

### Animal Experiments

#### Biosafety and Ethics Statements

*Brucella* and GMOs were used in the registered BSL3 facilities (code A/ES/15/I-05) of the Instituto de Agrobiotecnologiìa (IdAB), previous authorization of the Spanish Consejo Interministerial de Organismos Modificados Genéticamente (CIOMG) (A/ES/16/39). BALB/c 7-week-old female or CD1 4-week-old male and female mice were purchased from Charles River (Elbeuf, France) and accommodated for 2 weeks in the authorized IdAB animal facilities (ES/31-2016-000002-CR-SU-US) before experimental proceedings. The animals were kept in biosafety cages with water and food *ad libitum* and manipulations carried out following FELASA (Rehbinder et al., 2000) and ARRIVE (Percie du Sert et al., 2020) guidelines. Procedures were based on brucellosis standards (Grilló et al., 2012) and authorized by Gobierno de Navarra (PI-025-14) in compliance with the current Spanish (RD 53/2013; ECC/566/2015) and European (Directive UE 2010/63) legislations. Churra ewes were purchased from *B. melitensis* and *B. ovis* free herds of Castilla y León, Spain, and kept in the authorized BSL2 and BSL3 facilities of the Universidad Complutense de Madrid (UCM; ES/281200000147 and ES/280790000154). Before starting the experiment, all ewes were serologically assessed as free from the main reproductive infections (i.e., *Chlamydia abortus*, *Coxiellaburnetii*, *Salmonella* Abortusovis, *Toxoplama gondii*, and Maedi-Visna). The GMOs and animal experiments were evaluated by the ethics and biosecurity committee of UCM [OH (CEA)-UCM-32-2018], CIOMG (B/ES/18/31; A/ES/20/83), and Agencia Española de Medicamentos y Productos Sanitarios (AEMPS; 194/PIV and 432/ECV). Eventual authorizations were granted by Gobierno de la Comunidad de Madrid (PROEX 187/18). Note that the use of *Brucella* Gm^R^ strains is no longer under restricted use since 2014, as it does not compromise the control of disease in humans or animals (Smith et al., 2015).

#### Spleen Infection and Cytokine Profiles in Mice

In general, the mice were inoculated IP or SC with 0.1 ml of bacterial suspension and killed by cervical dislocation at selected intervals to determine the number of viable CFU/spleen (expressed as log_10_ CFU/spleen), and the spleens weight (grams/spleen), as reported (Grilló et al., 2006b). The mice inoculated with Rev1 in standard conditions (10^5^ CFU, IP or SC) or with PBS were used as controls.

A dose-response experiment was performed in the BALB/c mice inoculated IP with 10^5^, 10^6^, 10^7^ or 10^8^ CFU/mouse of Rev1Δ*wzm*, and the spleen counts/weights (*n* = 5) were determined at 2 weeks PI. Thereafter, with the two selected doses (10^6^ and 10^8^ CFU/mouse), we performed an experiment of persistence by determining the spleen counts and weights at 1, 2, 4, and/or 6 weeks PI.

For the three mutants, the kinetics of infection, weights, and cytokines in spleens were studied in groups of 14 BALB/c mice IP, inoculated with 10^8^ CFU/animal (selected as optimal dose) and weekly killed (*n* = 3 at 1 and 4 weeks PI; *n* = 4 at 2 and 3 weeks PI). For cytokines, individual supernatants were obtained (1,000 × *g*, 10 min) in Hanks balanced salt solution (Gibco) treated (1 h, 4°C) with 1% CHAPS (Sigma-Aldrich, San Luis, MO, United States) and filtrated (0.20 μm, Millipore). Moreover, blood samples were collected at 2, 6, and 24 h PI (*n* = 5) by retro-orbital plexus puncture in mice anesthetized (ISOFLO, Ecuphar) and processed as a pool (by mixing equal volume of serum from each mouse) by analyzing each pool in duplicate; Rev1Δ*wzm*, Rev1, and PBS groups (*n* = 5) were analyzed in two independent experiments. Similarly, the blood samples were obtained one time a week directly prior to each necropsy and processed individually (*n* = 5). Sera samples and splenocytes supernatants were used to determine IL-6, IL-12p40, TNF-α, and IFN-γ using commercial ELISA kits (BD OptEIA) in two technical replicates/sample. The results were expressed as pg/spleen and pg/ml of serum.

The *in vivo* stability of Rev1Δ*wzm* was evaluated after 5 serial passages in the mice (Schurig et al., 1991). Groups of 3 CD1 mice were inoculated IP at 10^8^ CFU/animal and necropsied at 3 days PI to determine the CFU/spleens. The bacteria recovered in each passage were subcultured to prepare the inocula of the next one. The genetic and phenotypic stability of the bacteria recovered from the last passage was assessed as described for the *in vitro* stability.

#### Vaccine Efficacy Studies

The efficacy of Rev1 *wzm*/*wzt* mutants to protect against *B. melitensis* or *B. ovis* virulent infections was analyzed in the BALB/c mice (*n* = 5), as reported (González et al., 2008; Soler-Lloréns et al., 2014). The mice (*n* = 5) were vaccinated IP or SC with 10^8^ CFU of the mutant and, 4 weeks later, were challenged IP with 10^4^ CFU of *B. melitensis* H38:Gm or with 5 × 10^5^ CFU of *B. ovis* PA:Gm. Additional groups (*n* = 5) of Rev1 in standard conditions (10^5^ CFU/animal, SC) and PBS were included as controls. The log_10_ CFU/spleen of the challenge strain was determined in BAB-Gm_15_, or in BAB-S-Gm_15_ with incubation in 10% CO_2_ at 2 or 3 weeks after challenging with H38 or *B. ovis* PA, respectively.

#### Safety in Pregnant Mice

Pregnancies were synchronized in the CD1 mice by light/darkness control and naturally mating for 2 days. On the day of pregnancy 4.5 ± 1, the mice were inoculated IP with 1 × 10^7^ CFU/mouse of Rev1Δ*wzm* (*n* = 14), or 1 × 10^6^CFU/mouse of Rev1 (*n* = 16) as control; 14 days later, all were necropsied to individually collect spleens, placentas, and fetuses. The CFU/g was determined by plating serial 10-fold PBS dilutions in BAB, applying external ethanol to avoid fetus-placenta cross contamination. For histopathological studies, placental tissues were fixed with 10% neutral buffered formaldehyde and stained with hematoxylin-eosin (H-E) (PanReac AppliChem, Castellar del Vallés, Barcelona, Spain).

#### Safety in Pregnant Ewes

Churra ewes (*n* = 6) were SC vaccinated on the day of gestation 75 ± 2 (DG75) with 1.8 × 10^10^ CFU of Rev1Δ*wzm*, including a PBS group of pregnant ewes (*n* = 4) as control. Clinical symptoms, rectal temperature, and local reactions were assessed during the first 2 weeks PI. Vaginal shedding was weekly monitored along the pregnancy by double-swab sampling and at parturition by collecting cotyledons and milk. The swabs were analyzed by (i) direct DNA extract and qRT-PCR of the IS*711* (Pérez-Sancho et al., 2013) and (ii) duplicate culturing in a CITA-selective medium (De Miguel et al., 2011) standard or modified by halving vancomycin and colistin concentrations, and incubation at 37°C for 7–14 days; presumptive colonies were confirmed by over-colony PCR with F1-R4*wzm* (Supplementary Table 2). Cotyledons homogenized in sterile PBS (1:10, w:v) and the milk samples were cultured (1 ml/plate) in these media. Within 4 weeks after delivery, all ewes and lambs were necropsied to determine bacterial presence in organs (spleen, liver, uterus, and mammary gland) and lymph nodes (pre-scapular, parotid, retropharyngeal, submaxillary, crural, iliac, and supra-mammary) by homogenization. Serological responses were weekly monitored in serum by an INgezim *B. ovis* kit (ELISA-R/LPS; Ingenasa), standard Rose Bengal test (RBT), and those reacting in RBT by S-LPS Complement Fixation test (CFT) at the officially accredited Laboratorio de Calidad Agroalimentaria de Navarra (Villava, Navarra, Spain), as recommended (OIE, 2018).

### Statistical Analysis

Statistical analysis and graphical representations were performed with GraphPad Prism 8 software (Inc., San Diego, CA, United States). *P*-values were determined by unpaired two-tailed Student’s *t*-test or by one- or two-way ANOVA, followed by Fisher’s least significant difference (LSD) test, with 95% confidence intervals, according to data classification. For PG analysis, only variations higher than 10% were considered as significant. The final figures were assembled using Adobe Illustrator 2020 (San José, CA, United States).

## Data Availability Statement

The original contributions presented in this study are included in the article/Supplementary Material, further inquiries can be directed to the corresponding author.

## Ethics Statement

Mice studies were reviewed and approved by Comité de Ética, Experimentación Animal y Bioseguridad (CEEAB) of Public University of Navarra (UPNA), Comité de Ética of CSIC, and the competent authority of Navarra Government; and sheep experiments were approved by the Ethics and Biosecurity Committee of Universidad Complutense de Madrid (UCM) and the competent authority of Comunidad de Madrid.

## Author Contributions

MG conceived, led, and supervised the study. SM-B designed and performed experiments. IP-U performed experiments in cells and pregnant mice. SM-B and MG carried out other mice experiments, and wrote the draft and the final manuscript. SM-B, IP-U, and MG participated in the sheep assay. SM-B, AZ-B, and LP contributed to mutants’ construction and to design some *in vitro* experiments. OI and FC performed UPLC-MS, and analyzed and discussed the results. SM-B, IP-U, AZ-B, and MG analyzed, discussed, and interpreted all results. All the authors revised and approved the final document for publication.

## Conflict of Interest

We would like to state that Rev1Δ*wzm* is protected by the patent WO/2019/101993 (PCT/EP2018/082539) belonging to CSIC and UPNA. The authors declare that the research was conducted in the absence of any commercial or financial relationships that could be construed as a potential conflict of interest.

## Publisher’s Note

All claims expressed in this article are solely those of the authors and do not necessarily represent those of their affiliated organizations, or those of the publisher, the editors and the reviewers. Any product that may be evaluated in this article, or claim that may be made by its manufacturer, is not guaranteed or endorsed by the publisher.

## Footnotes

1
https://brucellosisvaccine.org

2
www.gimp.org

## References

[B1] Alton G. G. Jones L. Angus R. Verger J. (1988). *Techniques for the Brucellosis Laboratory.* Paris: Institut National de la Recherche Agronomique.

[B2] Altschul S. F. Gish W. Miller W. Myers E. W. Lipman D. J. (1990). Basic local alignment search tool. *J. Mol. Biol.* 215 403–10. 10.1016/S0022-2836(05)80360-2 2231712

[B3] Alvarez L. Cordier B. van Teeffelen S. Cava F. (2020). Analysis of Gram-negative bacteria peptidoglycan by ultra-performance liquid chromatography. *Bio-Protocol* 10 1–14. 10.21769/bioprotoc.3780 33659436 PMC7842696

[B4] Alvarez L. Hernandez S. B. De Pedro M. A. Cava F. (2016). Ultra-sensitive, high-resolution liquid chromatography methods for the high-throughput quantitative analysis of bacterial cell wall chemistry and structure. *Methods Mol. Biol.* 1440 11–27. 10.1007/978-1-4939-3676-2_2 27311661

[B5] Aragón-Aranda B. De Miguel M. J. Lázaro-Antón L. Salvador-Bescós M. Zúñiga-Ripa A. Moriyón I. (2020). Development of attenuated live vaccine candidates against swine brucellosis in a non-zoonotic *B. suis* biovar 2 background. *Vet. Res.* 51:8. 10.1186/s13567-020-00815-8 32703299 PMC7376850

[B6] Arellano-Reynoso B. Lapaque N. Salcedo S. Briones G. Ciocchini A. E. Ugalde R. (2005). Cyclic β-1,2-glucan is a *Brucella* virulence factor required for intracellular survival. *Nat. Immunol.* 6 618–25. 10.1038/ni1202 15880113

[B7] Ariza J. Bosch J. Gudiol F. Liñares J. Viladrich P. F. Martín R. (1986). Relevance of *in vitro* antimicrobial susceptibility of *Brucella melitensis* to relapse rate in human brucellosis. *Antimicrob. Agents Chemother.* 30 958–60. 10.1128/AAC.30.6.958 3813520 PMC180631

[B8] Atun G. Hisarli G. Tunçay M. (1998). Adsorption of safranine-O on hydrophilic and hydrophobic glass surfaces. *Colloids Surfaces A Physicochem. Eng. Asp.* 143 27–33. 10.1016/S0927-7757(98)00494-4

[B9] Avila-Calderón E. D. Medina-Chávez O. Flores-Romo L. Hernández-Hernández J. M. Donis-Maturano L. López-Merino A. (2020). Outer membrane vesicles from *Brucella melitensis* modulate immune response and induce cytoskeleton rearrangement in peripheral blood mononuclear cells. *Front. Microbiol.* 11:1–18. 10.3389/fmicb.2020.556795 33193138 PMC7604303

[B10] Baldwin C. L. Goenka R. (2006). Host immune responses to the intracellular bacteria *Brucella*: does the bacteria instruct the host to facilitate chronic infection? *Crit. Rev. Immunol.* 26 407–42. 10.1615/CritRevImmunol.v26.i5.30 17341186

[B11] Barrio M. B. Grilló M. J. Muñoz P. M. Jacques I. González D. De Miguel M. J. (2009). Rough mutants defective in core and O-polysaccharide synthesis and export induce antibodies reacting in an indirect ELISA with smooth lipopolysaccharide and are less effective than Rev 1 vaccine against *Brucella melitensis* infection of sheep. *Vaccine* 27 1741–9. 10.1016/j.vaccine.2009.01.025 19186196

[B12] Bi Y. Zimmer J. (2020). Structure and ligand-binding properties of the O antigen ABC transporter carbohydrate-binding domain. *Structure* 28 252.e–8.e. 10.1016/j.str.2019.11.020 31879128 PMC7004846

[B13] Bi Y. Mann E. Whitfield C. Zimmer J. (2018). Architecture of a channel-forming O-antigen polysaccharide ABC transporter. *Nature* 553 361–5. 10.1038/nature25190 29320481 PMC5978415

[B14] Blasco J. M. (1997). A review of the use of *B. melitensis* Rev 1 vaccine in adult sheep and goats. *Prev. Vet. Med.* 31 275–83. 10.1016/s0167-5877(96)01110-5 9234451

[B15] Blasco J. M. Díaz R. (1993). *Brucella melitensis* Rev-1 vaccine as a cause of human brucellosis. *Lancet* 342:805. 10.1016/0140-6736(93)91571-3 8103891

[B16] Bosseray N. Plommet M. (1990). *Brucella suis* S2, *Brucella melitensis* Rev. 1 and *Brucella abortus* S19 living vaccines: residual virulence and immunity induced against three *Brucella* species challenge strains in mice. *Vaccine* 8 462–8. 10.1016/0264-410X(90)90247-J 2123586

[B17] Briones G. Iñón de Iannino N. Roset M. Vigliocco A. Silva Paulo P. Ugalde R. A. (2001). *Brucella abortus* cyclic β-1,2-glucan mutants have reduced virulence in mice and are defective in intracellular replication in HeLa cells. *Infect. Immun.* 69 4528–35. 10.1128/IAI.69.7.4528-4535.2001 11401996 PMC98529

[B18] Caffalette C. A. Zimmer J. (2021). Cryo-EM structure of the full-length Wzm/Wzt ABC transporter required for lipid-linked O antigen transport. *Proc. Natl. Acad. Sci. U S A.* 118 1–10. 10.1073/pnas.2016144118 33443152 PMC7817213

[B19] Caffalette C. A. Kuklewicz J. Spellmon N. Zimmer J. (2020). Biosynthesis and export of bacterial glycolipids. *Annu. Rev. Biochem.* 89 741–68. 10.1146/annurev-biochem-011520-104707 32569526

[B20] Caro-Hernández P. Fernández-Lago L. De Miguel M. J. Martín-Martín A. I. Cloeckaert A. Grilló M. J. (2007). Role of the Omp25/Omp31 family in outer membrane properties and virulence of *Brucella ovis*. *Infect. Immun.* 75 4050–61. 10.1128/IAI.00486-07 17562767 PMC1952020

[B21] Castañeda-Roldán E. I. Avelino-Flores F. Dall’Agnol M. Freer E. Cedillo L. Dornand J. (2004). Adherence of *Brucella* to human epithelial cells and macrophages is mediated by sialic acid residues. *Cell. Microbiol.* 6 435–45. 10.1111/j.1462-5822.2004.00372.x 15056214

[B22] Cheung C. Lee J. Lee J. Shevchuk O. (2009). The effect of ionic (NaCl) and non-ionic (sucrose) osmotic stress on the expression of β-galactosidase in wild type *E.coli* BW25993 and in the isogenic BW25993Δ*lacI* mutant. *J. Exp. Microbiol. Immunol.* 13 1–6.

[B23] Clarke B. R. Cuthbertson L. Whitfield C. (2004). Nonreducing terminal modifications determine the chain length of polymannose O antigens of *Escherichia coli* and couple chain termination to polymer export *via* an ATP-binding cassette transporter. *J. Biol. Chem.* 279 35709–18. 10.1074/jbc.M404738200 15184370

[B24] Clifton L. A. Ciesielski F. Skoda M. W. A. Paracini N. Holt S. A. Lakey J. H. (2016). The effect of lipopolysaccharide core oligosaccharide size on the electrostatic binding of antimicrobial proteins to models of the Gram-negative bacterial outer membrane. *Langmuir* 32 3485–94. 10.1021/acs.langmuir.6b00240 27003358 PMC4854487

[B25] Conde-Álvarez R. Arce-Gorvel V. Iriarte M. Manček-Keber M. Barquero-Calvo E. Palacios-Chaves L. (2012). The lipopolysaccharide core of *Brucella abortus* acts as a shield against innate immunity recognition. *PLoS Pathog.* 8:e1002675. 10.1371/journal.ppat.1002675 22589715 PMC3349745

[B26] Cuthbertson L. Kimber M. S. Whitfield C. (2007). Substrate binding by a bacterial ABC transporter involved in polysaccharide export. *Proc. Natl. Acad. Sci.* 104 19529–34. 10.1073/pnas.0705709104 18032609 PMC2148323

[B27] Cuthbertson L. Powers J. Whitfield C. (2005). The C-terminal domain of the nucleotide-binding domain protein Wzt determines substrate specificity in the ATP-binding cassette transporter for the lipopolysaccharide O-antigens in *Escherichia coli* serotypes O8 and O9a. *J. Biol. Chem.* 280 30310–9. 10.1074/jbc.M504371200 15980069

[B28] Czerwonka G. Guzy A. Kałuża K. Grosicka M. Dańczuk M. Lechowicz Ł (2016). The role of *Proteus mirabilis* cell wall features in biofilm formation. *Arch. Microbiol.* 198 877–84. 10.1007/s00203-016-1249-x 27262948 PMC5040740

[B29] De Miguel M. J. Marín C. M. Muñoz P. M. Dieste L. Grilló M. J. Blasco J. M. (2011). Development of a selective culture medium for primary isolation of the main *Brucella* species. *J. Clin. Microbiol.* 49 1458–63. 10.1128/JCM.02301-10 21270216 PMC3122841

[B30] Desmarais S. M. De Pedro M. A. Cava F. Huang K. C. (2013). Peptidoglycan at its peaks: how chromatographic analyses can reveal bacterial cell wall structure and assembly. *Mol. Microbiol.* 89 1–13. 10.1111/mmi.12266 23679048 PMC3694805

[B31] Dohnalkova A. C. Marshall M. J. Arey B. W. Williams K. H. Buck E. C. Fredrickson J. K. (2011). Imaging hydrated microbial extracellular polymers: comparative analysis by electron microscopy. *Appl. Environ. Microbiol.* 77 1254–62. 10.1128/AEM.02001-10 21169451 PMC3067245

[B32] Dong H. Liu W. Peng X. Wu Q. (2015). The effects of RegM on stress responses in *Brucella melitensis*. *Curr. Microbiol.* 70 730–4. 10.1007/s00284-015-0782-1 25648428

[B33] Dorneles E. M. S. Teixeira-Carvalho A. Araújo M. S. S. Sriranganathan N. Lage A. P. (2015). Immune response triggered by *Brucella abortus* following infection or vaccination. *Vaccine* 33 3659–66. 10.1016/j.vaccine.2015.05.057 26048781

[B34] EUCAST: European Committee for Antimicrobial Susceptibility Testing. (2003). *Determination of Minimum Inhibitory Concentrations (MICs) of Antibacterial Agents by Broth Dilution.* Central Hospital Växjö: EUCAST.

[B35] Fletcher M. Loeb G. I. (1979). Influence of substratum characteristics on the attachment of a marine *Pseudomonas* to solid surfaces. *Appl. Environ. Microbiol.* 37 67–72. 10.1128/aem.37.1.67-72.1979 16345338 PMC243402

[B36] Fontana C. Conde-Álvarez R. Ståhle J. Holst O. Iriarte M. Zhao Y. (2016). Structural studies of lipopolysaccharide-defective mutants from *Brucella melitensis* identify a core oligosaccharide critical in virulence. *J. Biol. Chem.* 291 7727–41. 10.1074/jbc.M115.701540 26867577 PMC4817197

[B37] Gamazo C. Moriyón I. (1987). Release of outer membrane fragments by exponentially growing *Brucella melitensis* cells. *Infect. Immun.* 55 609–15. 10.1128/iai.55.3.609-615.1987 3818086 PMC260382

[B38] Godefroid M. Svensson M. V. Cambier P. Uzureau S. Mirabella A. De Bolle X. (2010). *Brucella melitensis* 16M produces a mannan and other extracellular matrix components typical of a biofilm. *FEMS Immunol. Med. Microbiol.* 59 364–77. 10.1111/j.1574-695X.2010.00689.x 20497223

[B39] Godessart P. Lannoy A. Dieu M. Van der Verren S. E. Soumillion P. Collet J. F. (2021). β-barrels covalently link peptidoglycan and the outer membrane in the α-proteobacterium *Brucella abortus*. *Nat. Microbiol.* 6 27–33. 10.1038/s41564-020-00799-3 33139884

[B40] Godfroid F. Cloeckaert A. Taminiau B. Danese I. Tibor A. De Bolle X. (2000). Genetic organisation of the lipopolysaccharide O-antigen biosynthesis region of *Brucella melitensis* 16M (*wbk*). *Res. Microbiol.* 151 655–68. 10.1016/s0923-2508(00)90130-x 11081580

[B41] Goldberg J. B. (1999). *Genetics of Bacterial Polysaccharides*, 1st Edn. Boca Raton, FL: CRC Press LLC, 10.1201/9781420074413

[B42] González D. Grilló M. J. De Miguel M. J. Ali T. Arce-Gorvel V. Delrue R. M. (2008). Brucellosis vaccines: assessment of *Brucella melitensis* lipopolysaccharide rough mutants defective in core and O-polysaccharide synthesis and export. *PLoS One* 3:e2760. 10.1371/journal.pone.0002760 18648644 PMC2453230

[B43] Grilló M. J. Blasco J. M. Gorvel J. Moriyón I. Moreno E. (2012). What have we learned from brucellosis in the mouse model? *Vet. Res.* 43 1–35. 10.1186/1297-9716-43-29 22500859 PMC3410789

[B44] Grilló M. J. Bosseray N. Blasco J. M. (2000). *In vitro* markers and biological activity in mice of seed lot strains and commercial *Brucella melitensis* Rev 1 and *Brucella abortus* B19 vaccines. *Biologicals* 28 119–27. 10.1006/biol.2000.0249 10885618

[B45] Grilló M. J. Manterola L. De Miguel M. J. Muñoz P. M. Blasco J. M. Moriyón I. (2006b). Increases of efficacy as vaccine against *Brucella abortus* infection in mice by simultaneous inoculation with avirulent smooth *bvrS*/*bvrR* and rough *wbkA* mutants. *Vaccine* 24 2910–6. 10.1016/j.vaccine.2005.12.038 16439039

[B46] Grilló M. J. De Miguel M. J. Muñoz P. M. Marín C. M. Ariza J. Blasco J. M. (2006a). Efficacy of several antibiotic combinations against *Brucella melitensis* Rev 1 experimental infection in BALB/c mice. *J. Antimicrob. Chemother.* 58 622–6. 10.1093/jac/dkl289 16849379

[B47] Grilló M. J. San Román Aberasturi B. Palacios Chaves L. Mena Bueno S. Zabalza Baranguá A. (2017). *A Modified Brucella Vaccine Strain for the Treatment of Brucellosis.* Madrid: Consejo Superior de Investigaciones Científicas.

[B48] Guidolin L. S. Morrone Seijo S. M. Guaimas F. F. Comerci D. J. Ciocchinia A. E. (2015). Interaction network and localization of *Brucella abortus* membrane proteins involved in the synthesis, transport, and succinylation of cyclic β-1,2-glucans. *J. Bacteriol.* 197 1640–8. 10.1128/JB.00068-15 25733613 PMC4403662

[B49] Guo R. Jiao Y. Li Z. Zhu S. Fei X. Geng S. (2017). Safety, protective immunity, and DIVA capability of a rough mutant *Salmonella* Pullorum vaccine candidate in broilers. *Front. Microbiol.* 8:1–10. 10.3389/fmicb.2017.00547 28424675 PMC5380749

[B50] Haag A. F. Myka K. K. Arnold M. F. F. Caro-Hernández P. Ferguson G. P. (2010). Importance of lipopolysaccharide and cyclic β-1,2-glucans in *Brucella*-mammalian infections. *Int. J. Microbiol.* 2010 1–12. 10.1155/2010/124509 21151694 PMC2995898

[B51] Hagelueken G. Clarke B. R. Huang H. Tuukkanen A. Danciu I. Svergun D. I. (2015). A coiled-coil domain acts as a molecular ruler to regulate O-antigen chain length in lipopolysaccharide. *Nat. Struct. Mol. Biol.* 22 50–6. 10.1038/nsmb.2935 25504321 PMC4650267

[B52] Hensel M. E. Garcia-Gonzalez D. G. Chaki S. P. Hartwig A. Gordy P. W. Bowen R. (2020). Vaccine candidate *Brucella melitensis* 16MΔ*vjbR* is safe in a pregnant sheep model and confers protection. *mSphere* 5:20. 10.1128/msphere.00120-20 32404509 PMC7227765

[B53] Hug I. Feldman M. F. (2011). Analogies and homologies in lipopolysaccharide and glycoprotein biosynthesis in bacteria. *Glycobiology* 21 138–51. 10.1093/glycob/cwq148 20871101

[B54] Issa M. N. Ashhab Y. (2016). Identification of *Brucella melitensis* Rev.1 vaccine-strain genetic markers: towards understanding the molecular mechanism behind virulence attenuation. *Vaccine* 34 4884–91. 10.1016/j.vaccine.2016.08.059 27595444

[B55] Izquierdo L. Merino S. Regué M. Rodriguez F. Tomás J. M. (2003). Synthesis of a *Klebsiella pneumoniae* O-antigen heteropolysaccharide (O12) requires an ABC 2 transporter. *J. Bacteriol.* 185 1634–41. 10.1128/JB.185.5.1634-1641.2003 12591881 PMC148082

[B56] Jain-Gupta N. Waldrop S. G. Tenpenny N. M. Witonsky S. G. Boyle S. M. Sriranganathan N. (2019). Rough *Brucella neotomae* provides protection against *Brucella suis* challenge in mice. *Vet. Microbiol.* 239:108447. 10.1016/j.vetmic.2019.108447 31767087

[B57] Jankowski A. Jankowski S. Mirończyk A. Niedbach J. (2005). The action of photosensitizers and serum in a bactericidal process. II. The effects of dyes: hypericin, eosin Y and saphranine O. *Polish J. Microbiol.* 54 323–30. 16599305

[B58] Jiménez de Bagüés M. P. Marín C. M. Barberán M. Blasco J. M. (1989). Responses of ewes to *B. melitensis* Rev1 vaccine administered by subcutaneous or conjunctival routes at different stages of pregnancy. *Ann. Rech. Vet.* 20 205–13. 2751232

[B59] Johnson Z. I. Chisholm S. W. (2004). Properties of overlapping genes are conserved across microbial genomes. *Genome Res.* 14 2268–72. 10.1101/gr.2433104 15520290 PMC525685

[B60] Kamens J. (2015). The Addgene repository: an international nonprofit plasmid and data resource. *Nucleic Acids Res.* 43 D1152–7. 10.1093/nar/gku893 25392412 PMC4384007

[B61] Kanehisa M. Goto S. (2000). KEGG: kyoto encyclopedia of genes and genomes. *Nucleic Acids Res.* 28 27–30. 10.1093/nar/28.1.27 10592173 PMC102409

[B62] Kohanski M. A. Dwyer D. J. Collins J. J. (2010). How antibiotics kill bacteria: from targets to networks. *Nat. Rev. Microbiol.* 8 423–35. 10.1038/nrmicro2333 20440275 PMC2896384

[B63] Kornspan D. Lubkovskaia R. Mathur S. Yeheskel A. Salmon-Divon M. (2020). Genomic analysis of natural rough *Brucella melitensis* Rev.1 vaccine strains: identification and characterization of mutations in key genes associated with bacterial lps biosynthesis and virulence. *Int. J. Mol. Sci.* 21 1–15. 10.3390/ijms21249341 33302421 PMC7762576

[B64] Kreutzer D. L. Scheffel J. W. Draper L. R. Robertson D. C. (1977). Mitogenic activity of cell wall components from smooth and rough strains of *Brucella abortus*. *Infect. Immun.* 15 842–5. 10.1128/iai.15.3.842-845.1977 404248 PMC421449

[B65] Kuo J. (2007). *Electron Microscopy: Methods and Protocols*, 2nd Edn. Totowa, NJ: Humana Press Inc, 10.1007/978-1-62703-776-1

[B66] Lalsiamthara J. Kaur G. Gogia N. Ali S. A. Goswami T. K. Chaudhuri P. (2020). *Brucella abortus* S19 *rfbD* mutant is highly attenuated, DIVA enable and confers protection against virulent challenge in mice. *Biologicals* 63 62–7. 10.1016/j.biologicals.2019.11.005 31843357

[B67] Lepage S. Lakaye B. Galleni M. Thamm I. Crine M. Groslambert S. (1995). Saturation of penicillin-binding protein 1 by β−lactam antibiotics in growing cells of *Bacillus licheniformis*. *Mol. Microbiol.* 16 365–72. 10.1111/j.1365-2958.1995.tb02308.x 7565098

[B68] Lerouge I. Laeremans T. Verreth C. Vanderleyden J. Van Soom C. Tobin A. (2001). Identification of an ATP-binding cassette transporter for export of the O-antigen across the inner membrane in *Rhizobium etli* based on the genetic, functional, and structural analysis of an LPS mutant deficient in O-antigen. *J. Biol. Chem.* 276 17190–8. 10.1074/jbc.M101129200 11279176

[B69] Livermore D. M. (1987). Mechanisms of resistance to cephalosporin antibiotics. *Drugs* 34 64–88. 10.2165/00003495-198700342-00007 3319506

[B70] Mancilla M. Grilló M. J. De Miguel M. J. López-Goñi I. San-Román B. Zabalza-Baranguá A. (2013). Deletion of the GI-2 integrase and the *wbkA* flanking transposase improves the stability of *Brucella melitensis* Rev 1 vaccine. *Vet. Res.* 44 1–12. 10.1186/1297-9716-44-105 24176078 PMC4176087

[B71] Martínez de Tejada G. Pizarro-Cerdá J. Moreno E. Moriyón I. (1995). The outer membranes of *Brucella* spp. are resistant to bactericidal cationic peptides. *Infect. Immun.* 63 3054–61. 10.1128/iai.63.8.3054-3061 7622230 PMC173416

[B72] Mirabella A. Terwagne M. Zygmunt M. S. Cloeckaert A. De Bolle X. Letesson J. J. (2013). *Brucella melitensis* MucR, an orthologue of *Sinorhizobium meliloti* MucR, is involved in resistance to oxidative, detergent, and saline stresses and cell envelope modifications. *J. Bacteriol.* 195 453–65. 10.1128/JB.01336-12 23161025 PMC3554010

[B73] Mohammad M. M. Tomita N. Ohta M. Movileanu L. (2016). The transmembrane domain of a bicomponent ABC transporter exhibits channel-forming activity. *ACS Chem. Biol.* 11 2506–18. 10.1021/acschembio.6b00383 27379442 PMC5026576

[B74] Monreal D. Grilló M. J. González D. Marín C. M. De Miguel M. J. López-Goñi I. (2003). Characterization of *Brucella abortus* O-polysaccharide and core lipopolysaccharide mutants and demonstration that a complete core is required for rough vaccines to be efficient against *Brucella abortus* and *Brucella ovis* in the mouse model. *Infect. Immun.* 71 3261–71. 10.1128/IAI.71.6.3261-3271.2003 12761107 PMC155776

[B75] Montaraz J. A. Winter A. J. Hunter D. M. Sowa B. A. Wu A. M. Adams L. G. (1986). Protection against *Brucella abortus* in mice with O-polysaccharide-specific monoclonal antibodies. *Infect. Immun.* 51 961–3. 10.1128/iai.51.3.961-963.1986 3005173 PMC260994

[B76] Moosavian M. Emam N. Pletzer D. Savari M. (2020). Rough-type and loss of the LPS due to *lpx* genes deletions are associated with colistin resistance in multidrug-resistant clinical *Escherichia coli* isolates not harbouring *mcr* genes. *PLoS One* 15:1–14. 10.1371/journal.pone.0233518 32433662 PMC7239443

[B77] Morè N. Martorana A. M. Biboy J. Otten C. Winkle M. Gurnani Serrano C. K. (2019). Peptidoglycan remodeling enables *Escherichia coli* to survive severe outer membrane assembly defect. *MBio* 10 e2729–2718. 10.1128/mBio.02729-18 PMC6428754 30723128

[B78] Moriyón I. Grilló M. J. Monreal D. González D. Marín C. López-Goñi I. (2004). Rough vaccines in animal brucellosis: structural and genetic basis and present status. *Vet. Res.* 35 1–38. 10.1051/vetres:2003037 15099501

[B79] Nakao R. Ramstedt M. Wai S. N. Uhlin B. E. (2012). Enhanced biofilm formation by *Escherichia coli* LPS mutants defective in Hep biosynthesis. *PLoS One* 7:1–13. 10.1371/journal.pone.0051241 23284671 PMC3532297

[B80] Nikaido H. Vaara M. (1985). Molecular basis of bacterial outer membrane permeability. *N Y. State J. Med.* 49 1–32. 10.1128/mr.49.1.1-32.1985 2580220 PMC373015

[B81] OIE (2018). “Chapter 3.1.4: Brucellosis (*Brucella abortus*, *B. melitensis* and *B. suis*) (Infection with *B. abortus*, *B. melitensis* and *B. suis*)’,” in *Office International of Epizooties, World Organisation for Animal Health. Manual of Diagnostic Tests and Vaccines for Terrestrial Animals.* (Paris: World Organisation for Animal Health).

[B82] Percie du Sert N. Hurst V. Ahluwalia A. Alam S. Avey M. T. Baker M. (2020). The ARRIVE guidelines 2.0: updated guidelines for reporting animal research. *PLoS Biol.* 18:e3000410. 10.1371/journal.pbio.3000410 32663219 PMC7360023

[B83] Perevoshchikova I. V. Sorochkina A. I. Zorov D. B. Antonenko Y. N. (2009). Safranine O as a fluorescent probe for mitochondrial membrane potential studied on the single particle level and in suspension. *Biochem* 74 663–71. 10.1134/s000629790906011x 19645672

[B84] Pérez-Sancho M. García-Seco T. Arrogante L. García N. Martínez I. Diez-Guerrier A. (2013). Development and evaluation of an IS*711*-based loop mediated isothermal amplification method (LAMP) for detection of *Brucella* spp. on clinical samples. *Res. Vet. Sci.* 95 489–94. 10.1016/j.rvsc.2013.05.002 23714043

[B85] Peters K. Pazos M. Edoo Z. Hugonnet J. E. Martorana A. M. Polissi A. (2018). Copper inhibits peptidoglycan LD-transpeptidases suppressing β-lactam resistance due to bypass of penicillin-binding proteins. *Proc. Natl. Acad. Sci. U S A.* 115 10786–91. 10.1073/pnas.1809285115 30275297 PMC6196517

[B86] Pilizota T. Shaevitz J. W. (2013). Plasmolysis and cell shape depend on solute outer-membrane permeability during hyperosmotic shock in *E. coli*. *Biophys. J.* 104 2733–42. 10.1016/j.bpj.2013.05.011 23790382 PMC3686340

[B87] Popescu A. Doyle R. (1996). The Gram stain after more than a century. *Biotech. Histochem.* 71 145–51. 10.1016/S0246-0343(10)19912-2 8724440

[B88] Poveda-Urkixo I. Ramírez G. A. Grilló M.-J. (2022). Kinetics of placental infection by different smooth *Brucella* strains in mice. *Pathogens* 11 1–17. 10.3390/PATHOGENS11030279 35335603 PMC8955611

[B89] Quandt J. Hynes M. F. (1993). Versatile suicide vectors which allow direct selection for gene replacement in Gram-negative bacteria. *Gene* 127 15–21. 10.1016/0378-1119(93)90611-6 8486283

[B90] Rehbinder C. Alenius S. Bures J. De las Heras M. Greko C. Kroon P. S. (2000). FELASA recommendations for the health monitoring of experimental units of calves, sheep and goats - Report of the federation of European Laboratory Animal Science Associations (FELASA) Working Group on Animal Health. *Lab. Anim.* 34 329–50. 10.1258/002367700780387723 11072854

[B91] Rocchetta H. L. Lam J. S. (1997). Identification and functional characterization of an ABC transport system involved in polysaccharide export of A-band lipopolysaccharide in *Pseudomonas aeruginosa*. *J. Bacteriol.* 179 4713–24. 10.1128/jb.179.15.4713-4724.1997 9244257 PMC179316

[B92] Rosenberg M. Gutnick D. Rosenberg E. (1980). Adherence of bacteria to hydrocarbons: a simple method for measuring cell-surface hydrophobicity. *FEMS Microbiol. Lett.* 9 29–33. 10.1111/j.1574-6968.1980.tb05599.x

[B93] Roset M. S. Ciocchini A. E. Ugalde R. A. Iñón de Iannino N. (2006). The *Brucella abortus*cyclic β-1,2-glucan virulence factor is substituted with O-ester-linked succinyl residues. *J. Bacteriol.* 188 5003–13. 10.1128/JB.00086-06 16816173 PMC1539967

[B94] Roset M. S. Ibañez A. E. De Souza Filho J. A. Spera J. M. Minatel L. Oliveira S. C. (2014). *Brucella* cyclic B-1,2-glucan plays a critical role in the induction of splenomegaly in mice. *PLoS One* 9:1–9. 10.1371/journal.pone.0101279 24983999 PMC4077732

[B95] Ruiz-Palma M. del S. Avila-Calderón E. D. Aguilera-Arreola M. G. López-Merino A. Ruiz E. A. (2021). Comparative proteomic analysis of outer membrane vesicles from *Brucella suis*, *Brucella ovis*, *Brucella canis* and *Brucella neotomae*. *Arch. Microbiol.* 203 1611–26. 10.1007/s00203-020-02170-w 33432377 PMC7799404

[B96] Salcedo S. P. Chevrier N. Lacerda T. L. S. Ben Amara A. Gerart S. Gorvel V. A. (2013). Pathogenic brucellae replicate in human trophoblasts. *J. Infect. Dis.* 207 1075–83. 10.1093/infdis/jit007 23303808

[B97] Salmon-Divon M. Yeheskel A. Kornspan D. (2018). Genomic analysis of the original Elberg *Brucella melitensis* Rev.1 vaccine strain reveals insights into virulence attenuation. *Lab. Anim.* 9 1436–48. 10.1080/21505594.2018.1511677 30139304 PMC6141144

[B98] Salmon-Divon M. Zahavi T. Kornspan D. (2019). Transcriptomic analysis of the *Brucella melitensis* Rev.1 vaccine strain in an acidic environment: insights into virulence attenuation. *Front. Microbiol.* 10:1–12. 10.3389/fmicb.2019.00250 30837973 PMC6382750

[B99] Sancho P. Tejedor C. Sidhu-Muñoz R. S. Fernández-Lago L. Vizcaíno N. (2014). Evaluation in mice of *Brucella ovis* attenuated mutants for use as live vaccines against *B. ovis* infection. *Vet. Res.* 45 1–10. 10.1186/1297-9716-45-61 24898325 PMC4057616

[B100] Sayers E. W. Bolton E. E. Brister J. R. Canese K. Chan J. Comeau D. C. (2022). Database resources of the National Center for Biotechnology Information (NCBI). *Nucleic Acids Res.* 50 D20–6. 10.1093/nar/gkab1112 34850941 PMC8728269

[B101] Schindelin J. Arganda-Carreras I. Frise E. Kaynig V. Longair M. Pietzsch T. (2012). Fiji: an open-source platform for biological-image analysis. *Nat. Methods* 9 676–82. 10.1038/nmeth.2019 22743772 PMC3855844

[B102] Schurig G. G. Roop R. M. Bagchi T. Boyle S. Buhrman D. Sriranganathan N. (1991). Biological properties of RB51; a stable rough strain of *Brucella abortus*. *Vet. Microbiol.* 28 171–88. 10.1016/0378-1135(91)90091-S 1908158

[B103] Schurig G. G. Sriranganathan N. Corbel M. J. (2002). Brucellosis vaccines: past, present and future. *Vet. Microbiol.* 90 479–96. 10.1016/S0378-1135(02)00255-9 12414166

[B104] Schuster C. F. Wiedemann D. M. Kirsebom F. C. M. Santiago M. Walker S. Gründling A. (2020). High-throughput transposon sequencing highlights the cell wall as an important barrier for osmotic stress in methicillin resistant *Staphylococcus aureus* and underlines a tailored response to different osmotic stressors. *Mol. Microbiol.* 113 699–717. 10.1111/mmi.14433 31770461 PMC7176532

[B105] Scupham A. J. Triplett E. W. (1997). Isolation and characterization of the UDP-glucose 4’-epimerase-encoding gene, *galE*, from *Brucella abortus* 2308. *Gene* 202 53–9. 10.1016/s0378-1119(97)00453-8 9427545

[B106] Shabala L. Bowman J. Brown J. Ross T. McMeekin T. Shabala S. (2009). Ion transport and osmotic adjustment in *Escherichia coli* in response to ionic and non-ionic osmotica. *Environ. Microbiol.* 11 137–48. 10.1111/j.1462-2920.2008.01748.x 18793315

[B107] Singh D. R. Mohammad M. M. Patowary S. Stoneman M. R. Oliver J. A. Movileanu L. (2013). Determination of the quaternary structure of a bacterial ATP-binding cassette (ABC) transporter in living cells. *Integr. Biol.* 5 312–23. 10.1039/C2IB20218B 23223798 PMC3558595

[B108] Smith J. Gangadharan D. Weyant R. (2015). Review of restricted experiment requests, division of select agents and toxins, centers for disease control and prevention, 2006-2013. *Health Secur.* 13 307–16. 10.1089/hs.2015.0021 26347984 PMC4582684

[B109] Solanki K. S. Varshney R. Qureshi S. Thomas P. Singh R. Agrawal A. (2021). Non-infectious outer membrane vesicles derived from *Brucella abortus* S19Δ*per* as an alternative acellular vaccine protects mice against virulent challenge. *Int. Immunopharmacol.* 90:107148. 10.1016/j.intimp.2020.107148 33189614

[B110] Soler-Lloréns P. Gil-Ramírez Y. Zabalza-Baranguá A. Iriarte M. Conde-Álvarez R. Zúñiga-Ripa A. (2014). Mutants in the lipopolysaccharide of *Brucella ovis* are attenuated and protect against *B. ovis* infection in mice. *Vet. Res.* 45 1–11. 10.1186/s13567-014-0072-0 25029920 PMC4107470

[B111] Spink W. W. Anderson D. (1954). Experimental studies on the significance of endotoxin in the pathogenesis of brucellosis. *J. Clin. Invest.* 33 540–8. 10.1172/JCI102924 13152193 PMC1087268

[B112] Spink W. W. Hall J. W. Finstad J. Mallet E. (1962). Immunization with viable *Brucella* organisms. Results of a safety test in humans. *Bull. World Health Organ.* 26 409–19. 13915813 PMC2555657

[B113] Stranahan L. W. Arenas-Gamboa A. M. (2021). When the going gets rough: the significance of *Brucella* lipopolysaccharide phenotype in host-pathogen interactions. *Front. Microbiol.* 12:713157. 10.3389/fmicb.2021.713157 34335551 PMC8319746

[B114] Tsai C. M. Frasch C. E. (1982). A sensitive silver stain for detecting lipopolysaccharides in polyacrylamide gels. *Anal. Biochem.* 119 115–9. 10.1016/0003-2697(82)90673-X 6176137

[B115] Uzureau S. Godefroid M. Deschamps C. Lemaire J. De Bolle X. Letesson J. J. (2007). Mutations of the quorum sensing-dependent regulator VjbR lead to drastic surface modifications in *Brucella melitensis*. *J. Bacteriol.* 189 6035–47. 10.1128/JB.00265-07 17557825 PMC1952030

[B116] Vaara M. (1992). Agents that increase the permeability of the outer membrane. *Microbiol. Rev.* 56 395–411. 10.1093/jac/dkq040 1406489 PMC372877

[B117] Valvano M. A. (2003). Export of O-specific lipopolysaccharide. *Front. Biosci.* 8:452–71. 10.2741/1079 12700099

[B118] Valvano M. A. (2015). “Chapter 4. Genetics and biosynthesis of lipopolysaccharide,” in *Molecular Medical Microbiology*, 2nd Edn. (Amsterdam: Elsevier Ltd), 55–89. 10.1016/B978-0-12-397169-2.00004-4

[B119] Vassen V. (2018). *Polarity of Envelope Growth and Heterogeneity of the Outer Membrane of Brucella Abortus.* Ph. D. Thesis. Namur: University of Namur.

[B120] Wang X. Wang L. Lu T. Yang Y. Chen S. Zhang R. (2014a). Effects of partial deletion of the *wzm* and *wzt* genes on lipopolysaccharide synthesis and virulence of *Brucella abortus* S19. *Mol. Med. Rep.* 9 2521–7. 10.3892/mmr.2014.2104 24718931

[B121] Wang X. Yan G. M. Zhang R. Lang X. L. Yang Y. L. Li X. Y. (2014b). Immunogenic response induced by *wzm* and *wzt* gene deletion mutants from *Brucella abortus* S19. *Mol. Med. Rep.* 9 653–8. 10.3892/mmr.2013.1810 24247358

[B122] Wang Z. Niu J. R. Wang X. L. Wu T. L. Cheng J. Lu L. (2014). Evaluation of a *Brucella melitensis* mutant deficient in O-polysaccharide export system ATP-binding protein as a rough vaccine candidate. *Microbes Infect.* 16 633–9. 10.1016/j.micinf.2014.06.013 25043564

[B123] Whitfield C. (1995). Biosynthesis of lipopolysaccharide O antigens. *Trends Microbiol.* 3 178–85. 10.1016/s0966-842x(00)88917-9 7542987

[B124] Williams D. M. Ovchinnikova O. G. Koizumi A. Mainprize I. L. Kimber M. S. Lowary T. L. (2017). Single polysaccharide assembly protein that integrates polymerization, termination, and chain-length quality control. *Proc. Natl. Acad. Sci. U S A.* 114 E1215–23. 10.1073/pnas.1613609114 28137848 PMC5321029

[B125] Wolter D. J. Lister P. D. (2013). Mechanisms of β-lactam resistance among *Pseudomonas aeruginosa*. *Curr. Pharm. Des.* 19 209–22. 10.2174/1381612811306020209 22894618

[B126] Wood P. J. (1980). Specificity in the interaction of direct dyes with polysaccharides. *Carbohydr. Res.* 85 271–87. 10.1016/S0008-6215(00)84676-5

[B127] Zabalza Baranguá A. (2017). *Desarrollo De Vacunas Marcadas con GFP Frente a la Brucelosis Ovina y Tests Diagnósticos Asociados.* Ph. D. Thesis. Navarre: Public University of Navarre.

[B128] Zhao Y. Arce-Gorvel V. Conde-Álvarez R. Moriyón I. Gorvel J. P. (2018). Vaccine development targeting lipopolysaccharide structure modification. *Microbes Infect.* 20 455–60. 10.1016/j.micinf.2017.11.006 29233768

