# Supplementary material for: Brucella melitensis Wzm/Wzt System: Changes in the Bacterial Envelope Lead to Improved Rev1Δwzm Vaccine Properties
Source: Front Microbiol. 2022 Jul 4;13:908495. doi: 10.3389/fmicb.2022.908495 (PMC9306315; doi:10.3389/fmicb.2022.908495)
Supplement: Supplementary file 1 [file Image_1.pdf]

A **Dose-response**

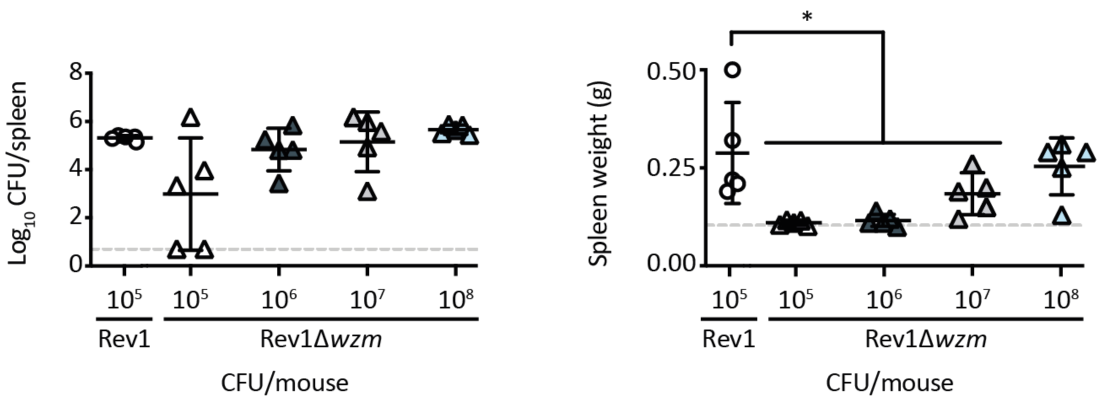

B **Splenic multiplication**

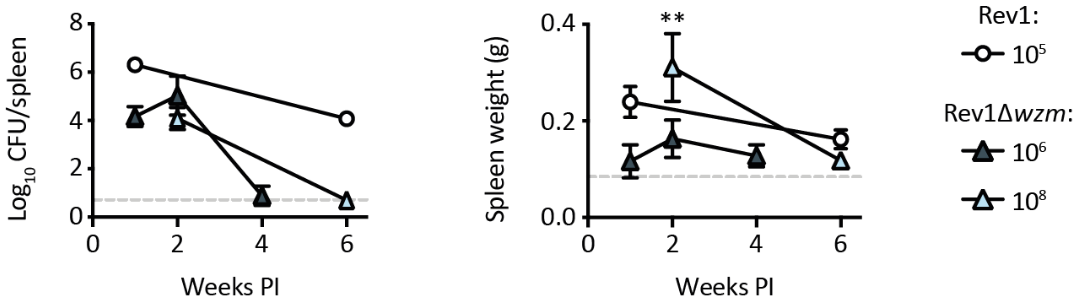

Supplementary Figure 1. Rev1Δ*wzm* is highly attenuated in BALB/c mice and the splenomegaly induced is dose dependent. **(A)** Dose-infection response experiment at 2 weeks PI, and **(B)** spleen growth curves studies for 6 weeks PI. BALB/c mice were inoculated IP at the indicated dose of Rev1Δ*wzm* or with 10^5 CFU/mouse of Rev1 as control and necropsied (n=5) at the indicated intervals to determine the mean±SD of $\log_{10}$ CFU/spleen and grams/spleen. Dashed lines indicate the detection limit and the normal weight of uninfected spleens (0.1 g). Statistical differences were determined by Fisher's LSD or t-tests: $^{**}p \leq 0.01$, $^{*}p \leq 0.05$.
