# Supplementary material for: Brucella melitensis Wzm/Wzt System: Changes in the Bacterial Envelope Lead to Improved Rev1Δwzm Vaccine Properties
Source: Front Microbiol. 2022 Jul 4;13:908495. doi: 10.3389/fmicb.2022.908495 (PMC9306315; doi:10.3389/fmicb.2022.908495)
Supplement: Supplementary file 2 [file Image_2.pdf]

HYPOTHESIS

Cell envelope pleiotropic modifications in *wzm/wzt* mutants

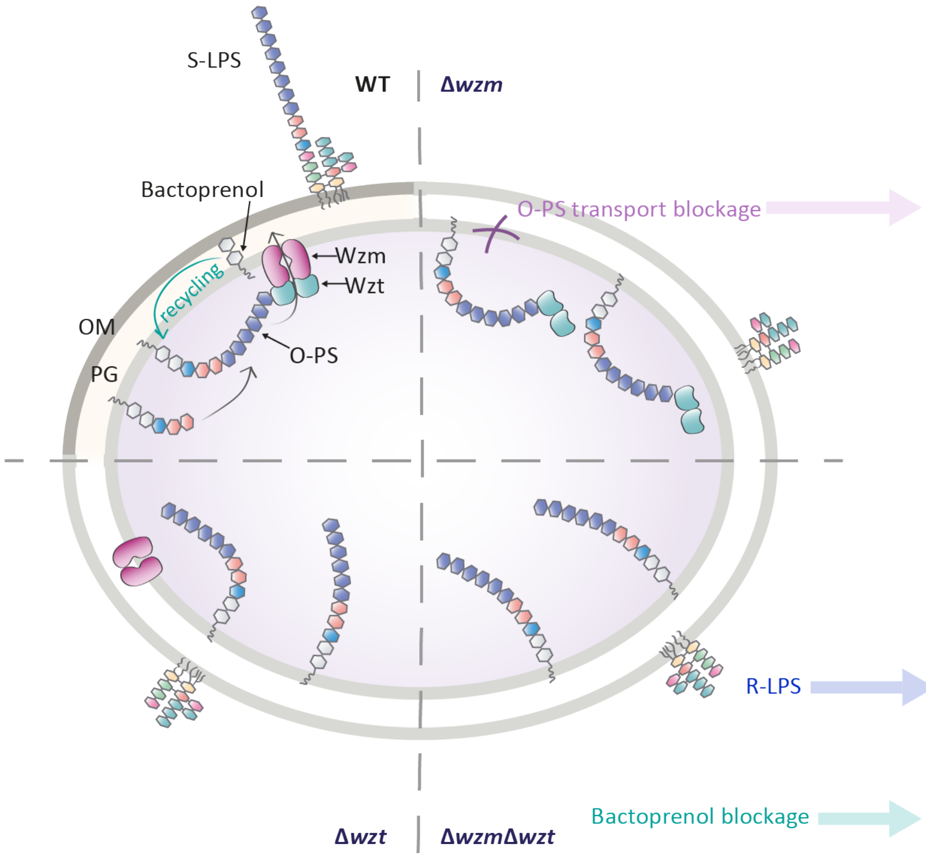

RESULTS

Rev1Δ*wzm*	Rev1Δ*wzt*	Rev1Δ*wzm*Δ*wzt*
No *wzm* expression	No *wzt* expression	No *wzm/wzt* expression
***wzt* overexpression**	*wzm* normal expression	

Antigenicity

wbkB & *wbkC* upregulation
Cytoplasmic O-PS & complete core
Susceptibility to environmental factors
Susceptibility to soluble factors of the immune system
High attenuation in mice spleens

Splenomegaly
Cytokine balance
B. ovis killing by immune serum
Efficacy vs. challenges
Genetic/phenotypic stability
Attenuation in BeWo cells
Safety in pregnant mice
Safety in pregnant ewes
Minimal serological interference

OM

Susceptibility to antibiotics (polymyxins, doxiciclin, rifampicin, gentamicin & streptomycin) and to cationic dyes (safranin O) • Autoagglutination • Adhesion to surfaces
Increased hydrophobicity • Negative bacterial charge • Blebs overproduction
Susceptibility to hyperosmotic stress

Cell wall

Relative resistance to low doses of penicillin G • High susceptibility to vancomicyn
1st description of *Brucella*'s PG stem composition
Decreased amount of PG • Reduction of L,D-crosslink

Supplementary Figure 2. Schematic representation of the starting hypothesis (left) and the main results obtained (right) concerning bacterial envelope changes associated with antigenicity, OM and cell wall in Rev1 *wzm/wzt* mutants.
