# Supplementary material for: Brucella melitensis Wzm/Wzt System: Changes in the Bacterial Envelope Lead to Improved Rev1Δwzm Vaccine Properties
Source: Front Microbiol. 2022 Jul 4;13:908495. doi: 10.3389/fmicb.2022.908495 (PMC9306315; doi:10.3389/fmicb.2022.908495)
Supplement: Supplementary file 3 [file Table_1.pdf]

SUPPLEMENTARY INFORMATION

Supplementary Table 1. Bacterial strains used in this work

Brucella	**Description and characteristics**	**Source/Reference**
Rev1	*B. melitensis* Rev1 reference vaccine parental; P_5^s, $Str_{2.5}^r$, S-LPS	IdAB collection
Rev1Δ*wzm*	Rev1 in-frame deletion mutant in *wzm* (Δ27-241); R-LPS	This work
Rev1Δ*wzt*	Rev1 in-frame deletion mutant in *wzt* (Δ68-209); R-LPS	
Rev1Δ*wzm*Δ*wzt*	Rev1 in-frame deletion mutant firstly in *wzm,* and secondly in *wzt*; R-LPS	
Rev1-NM	Rev1 strains non-mutated after *wzm* or *wzt* allelic exchange; S-LPS	
Rev1Δ*wzm*-NM	Rev1Δ*wzm* non-mutated strain after *wzt* allelic exchange during Rev1Δ*wzm*Δ*wzt* construction; R-LPS	
Rev1Δ*wzm*-pSRK*wzm*	Rev1Δ*wzm* complemented with the non-integrative vector pSRK*wzm*; Km_{50}^r, S-LPS	
Rev1Δ*wzt*-pSRK*wzt*	Rev1Δ*wzt* complemented with the non-integrative vector pSRK*wzt*; Km_{50}^r, S-LPS	
Rev1Δ*wzm*Δ*wzt*-pSRK*wzm-wzt*	Rev1Δ*wzm*Δ*wzt* complemented with the non-integrative vector pSRK*wzm-wzt*; Km_{50}^r, S-LPS	
16M	*B. melitensis* bv. 1 reference virulent strain; S-LPS	IdAB collection
16MΔ*wzm*	16M in-frame deletion mutant in *wzm*; R-LPS	Zabalza-Baranguá, 2017
BmH38R*manB*$_{core}$	H38 carrying the mini-Tn*5*-Km in *manB*$_{core}$ (H38::Tn*5*-manB$_{core}$); Nal_{25}^r, Km_{50}^r, R-LPS and incomplete core	González et al., 2008
H38::Gm	*B. melitensis* bv. 1 carrying mini-Tn*7*-*gfp*-Gm; Nal_{25}^r, Gm_{15}^r, S-LPS	IdAB collection
BoPA	*B. ovis* strain PA reference virulent strain; R-LPS	
BoPA::Gm	BoPA strain carrying mini-Tn*7*-*gfp*-Gm; Nal_{25}^r, Gm_{15}^r, R-LPS	
2308	*B. abortus* bv. 1 reference strain; S-LPS	
***E. coli* - plasmid constructions**	**Description and characteristics**	**Source/Reference**
S17λpir - pJQKmΔ*wzm*	Conjugative donor strain carrying BMEI1415 Δ27-241 into pJQKm suicide vector; *lac*, Km_{50}^r, Suc_5^s	Zabalza-Baranguá, 2017
S17λpir - pSRK*wzm*	Conjugative donor train with pSRK vector for Δ*wzm* mutant complementation; *lac*, Km_{50}^r	
TOP10F´- pCR2.1Δ*wzt*	Competent strain carrying BMEI1416 Δ68-209 into pCR2.1 cloning vector; *lac*, Km_{50}^r	This work
TOP10F'pJQKmΔ*wzt*	Competent strain carrying BMEI1416 Δ68-209 into pJQKm suicide vector; *lac*; Km_{50}^r, Suc_5^s	
S17λpir - pJQKmΔ*wzt*	Conjugative donor strain carrying BMEI1416 Δ68-209 into pJQKm suicide vector; *lac*, Km_{50}^r, Suc_5^s	
TOP10F'pCR2.1*wzt*	Competent strain carrying BMEI1416 ORF into pCR2.1 cloning vector; *lac*; Km_{50}^r	
TOP10F'pSRK*wzt*	Competent strain carrying BMEI1416 ORF into pSRK vector; *lac*; Km_{50}^r	
S17λpir - pSRK*wzt*	Conjugative donor strain with pSRK carrying BMEI1416 ORF for Δ*wzt* mutant complementation; *lac*, Km_{50}^r	
TOP10F'pCR2.1*wzm-wzt*	Competent strain carrying BMEI1415-16 ORFs into pCR2.1 cloning vector; *lac*; Km_{50}^r	
TOP10F'pSRK*wzm-wzt*	Competent strain carrying BMEI1415-16 ORFs into pSRK vector; *lac*; Km_{50}^r	
S17λpir - pSRK*wzm-wzt*	Conjugative strain with pSRK carrying BMEI1415-16 ORFs for Δ*wzm*Δ*wzt* mutant complementation; *lac*, Km_{50}^r	
E. coli K12	Reference Gram-negative laboratory strain, used as control in antimicrobial susceptibility studies	IdAB collection

LPS: lipopolysaccharide; S: smooth; R: rough; resistance to nalidixic acid (Nal_{25}^r), kanamycin (Km_{50}^r), streptomycin ($Str_{2.5}^r$), gentamycin (Gm_{15}^r) at the indicated dose (µg/mL); susceptibility to penicillin G (P_5^s), sucrose (Suc_5^s). *lac*: lactose operon.
