# Supplementary material for: Brucella melitensis Wzm/Wzt System: Changes in the Bacterial Envelope Lead to Improved Rev1Δwzm Vaccine Properties
Source: Front Microbiol. 2022 Jul 4;13:908495. doi: 10.3389/fmicb.2022.908495 (PMC9306315; doi:10.3389/fmicb.2022.908495)
Supplement: Supplementary file 4 [file Table_2.pdf]

Supplementary Table 2. Primers used for construction and genetic characterisation of *wzm*/*wzt* mutants

ORF (gene)	Primer	Hybridizing region	Sequence (5'→3')	Source/Reference
BMEI1415 (*wzm*)	F1	upstream	gcaaattgaaatggcagatg	Zabalza Barangúa, 2017
	R2	inner region	agcgcccacgtaaatcag	
	F3	complementary to R2 + inner region	ctgatttacgtgggcgcttaacctgcgtggcagtagc	
	R4	downstream	atgaaacgtggcgttagtcc	
	F7	start codon and upstream nucleotides	gtgagacgatttcgtatgata	This work
	F9	start codon	atgatatcgtatatggctaatg	
	R8	stop codon	tcataggtaaaaaatggctctc	
BMEI1416 (*wzt*)	F1	upstream	gttcaggcagggtggattga	This work
	R2	inner region	tgtgacctatcagggcaacg	
	F3	complementary to R2 + inner region	cgttgccctgataggtcacacaagccatgaccttgcgaac	
	R4	downstream	acagaagccggaatcgtcag	
	F6	start codon	atgatccagccatcgattaccc	
	R7	stop codon	tcatgctatagctcccattccc	
BMEI0993	P1 (F)	GI-2 region	caacatcgcaaagcctgaaa	Mancilla et al., 2013
BMEI1013	P2 (R)		cgcaatccagccaatacctg	
BMEI0994	P3 (R)		atcgtcggcattgtctctct	
BMEI1398	P5 (F)	*wbk* region	gatcttggtatcggcctgtc	
BMEI1413	P6 (R)		tgcgactttcttcacgattg	
BMEI1400	P7 (R)		cgctttaatatctcgcgttcc	

F: forward; R: reverse. PCR amplification with F9-R8 *wzm*: 783 bp (WT and Δ*wzt*) or 141 bp (Δ*wzm* and Δ*wzm*Δ*wzt*); F6-R7 *wzt*: 759 bp (WT and Δ*wzm*) or 336 bp (Δ*wzt* and Δ*wzm*Δ*wzt*); and F7 *wzm*-R7 *wzt*: 1553 bp (WT), 911 bp (Δ*wzm*), 1130 bp (Δ*wzt*) or 488 bp (Δ*wzm*Δ*wzt*).
