# Supplementary material for: Brucella melitensis Wzm/Wzt System: Changes in the Bacterial Envelope Lead to Improved Rev1Δwzm Vaccine Properties
Source: Front Microbiol. 2022 Jul 4;13:908495. doi: 10.3389/fmicb.2022.908495 (PMC9306315; doi:10.3389/fmicb.2022.908495)
Supplement: Supplementary file 5 [file Table_3.pdf]

Supplementary Table 3. Sequencing and alignment of *wzm-wzt* from Rev1, Rev1Δ*wzm*, Rev1Δ*wzt* and Rev1Δ*wzm*Δ*wzt*

Strain	Sequence (5´→3´)	bp
Rev1	GTGAGACGATTTCGTATGATATCGTATATGGCTAATGTCTGGAAGGTACGCCACTTCTGGTGGCACCTTTCAATGTCTGATTTACGTGGGCGCTTCAGGCGGTCCTCCTTGGGAATATTA	120
Rev1Δ*wzm*	GTGAGACGATTTCGTATGATATCGTATATGGCTAATGTCTGGAAGGTACGCCACTTCTGGTGGCACCTTTCAATGTCTGATTTACGTGGGCGCT--------------------------	94
Rev1Δ*wzt*	GTGAGACGATTTCGTATGATATCGTATATGGCTAATGTCTGGAAGGTACGCCACTTCTGGTGGCACCTTTCAATGTCTGATTTACGTGGGCGCTTCAGGCGGTCCTCCTTGGGAATATTA	120
Rev1Δ*wzm*Δ*wzt*	GTGAGACGATTTCGTATGATATCGTATATGGCTAATGTCTGGAAGGTACGCCACTTCTGGTGGCACCTTTCAATGTCTGATTTACGTGGGCGCT--------------------------	94
Rev1	TGGGCAGTTATACAGCCACTAGCGCTCACGCTGCTACTGTCTTTCGTGTTTTCTAAATTGTTGAATCAAAGTATATCTGCATATGCCCCCTATATTCTATCTGGGATTATTATCTGGGAA	240
Rev1Δ*wzm*	--	95
Rev1Δ*wzt*	TGGGCAGTTATACAGCCACTAGCGCTCACGCTGCTACTGTCTTTCGTGTTTTCTAAATTGTTGAATCAAAGTATATCTGCATATGCCCCCTATATTCTATCTGGGATTATTATCTGGGAA	240
Rev1Δ*wzm*Δ*wzt*	--	95
Rev1	TACATATCATTTACAGTGGTTGGTGGCTCAACAGCGCTTGTGCAAGCCGATGCATATATAAAGCAAACCAGAAATCCTCTTGCAATTTACACGCTTAGGAACACTGTTTCTGGCTTGGTC	360
Rev1Δ*wzm*	--	95
Rev1Δ*wzt*	TACATATCATTTACAGTGGTTGGTGGCTCAACAGCGCTTGTGCAAGCCGATGCATATATAAAGCAAACCAGAAATCCTCTTGCAATTTACACGCTTAGGAACACTGTTTCTGGCTTGGTC	360
Rev1Δ*wzm*Δ*wzt*	--	95
Rev1	GTATTATCCGTAGCAAGTATCTCCCTATTCGGGTGGGTACTTATCATGTTTCCTGAAAACTTCTCGCTTTCATGGTTAGCAATACCAACTTTGCTACCCATCCTTGCTTTGATAGTTTGG	480
Rev1Δ*wzm*	--	95
Rev1Δ*wzt*	GTATTATCCGTAGCAAGTATCTCCCTATTCGGGTGGGTACTTATCATGTTTCCTGAAAACTTCTCGCTTTCATGGTTAGCAATACCAACTTTGCTACCCATCCTTGCTTTGATAGTTTGG	480
Rev1Δ*wzm*Δ*wzt*	--	95
Rev1	CCGCTTGCCACAATCGTCGGCTACATCGGCGCAAGATTTCGAGATCTGCCGAATGCTCTGGCGCTCGTGTTACAGGCAGCTTGGTTTGTTTCGCCGGTCTATTTTAAAGAATCGATGTTC	600
Rev1Δ*wzm*	--	95
Rev1Δ*wzt*	CCGCTTGCCACAATCGTCGGCTACATCGGCGCAAGATTTCGAGATCTGCCGAATGCTCTGGCGCTCGTGTTACAGGCAGCTTGGTTTGTTTCGCCGGTCTATTTTAAAGAATCGATGTTC	600
Rev1Δ*wzm*Δ*wzt*	--	95
Rev1	AGGCAGGGTGGATTGAATGCATTCGTTGATTATAACCCTATTTACCACGTGATGCAGATTCTAAGAGCCCCTGTCCTTTATGGGGAATGGCCTACGGCTACCAATTACATTTGGTGCTTA	720
Rev1Δ*wzm*	--	95
Rev1Δ*wzt*	AGGCAGGGTGGATTGAATGCATTCGTTGATTATAACCCTATTTACCACGTGATGCAGATTCTAAGAGCCCCTGTCCTTTATGGGGAATGGCCTACGGCTACCAATTACATTTGGTGCTTA	720
Rev1Δ*wzm*Δ*wzt*	--	95
Rev1	GGTGTGAGCCTCCTCCTAACCTGCGTGGCAGTAGCTGTGGGGATGCGTGCGGAGAAGAGAGCCATTTTTTACCTATGATCCAGCCATCGATTACCCTGTCAAATGTTCATCTGCACTACG	840
Rev1Δ*wzm*	----------------TAACCTGCGTGGCAGTAGCTGTGGGGATGCGTGCGGAGAAGAGAGCCATTTTTTACCTATGATCCAGCCATCGATTACCCTGTCAAATGTTCATCTGCACTACG	198
Rev1Δ*wzt*	GGTGTGAGCCTCCTCCTAACCTGCGTGGCAGTAGCTGTGGGGATGCGTGCGGAGAAGAGAGCCATTTTTTACCTATGATCCAGCCATCGATTACCCTGTCAAATGTTCATCTGCACTACG	840
Rev1Δ*wzm*Δ*wzt*	----------------TAACCTGCGTGGCAGTAGCTGTGGGGATGCGTGCGGAGAAGAGAGCCATTTTTTACCTATGATCCAGCCATCGATTACCCTGTCAAATGTTCATCTGCACTACG	198

```
Rev1              CTGCATCAGCGTTCAAAGAACGCTCAGTCAAAACTCTAGTAAACGCCTTATTGAGTATGCGGCGCAGCGCAGGAGCAAACATTGAAGACATCCATGCCCTAAAGGGTATTTCTGTAGATA   960
Rev1Δwzm          CTGCATCAGCGTTCAAAGAACGCTCAGTCAAAACTCTAGTAAACGCCTTATTGAGTATGCGGCGCAGCGCAGGAGCAAACATTGAAGACATCCATGCCCTAAAGGGTATTTCTGTAGATA   318
Rev1Δwzt          CTGCATCAGCGTTCAAAGAACGCTCAGTCAAAACTCTAGTAAACGCCTTATTGAGTATGCGGCGCAGCGCAGGAGCAAACATTGAAGACATCCATGCCCTAAAGGGTATTTCTGTAGATA   960
Rev1ΔwzmΔwzt      CTGCATCAGCGTTCAAAGAACGCTCAGTCAAAACTCTAGTAAACGCCTTATTGAGTATGCGGCGCAGCGCAGGAGCAAACATTGAAGACATCCATGCCCTAAAGGGTATTTCTGTAGATA   318

Rev1              TAGCGCGGGGCGAACGCGTTGCCCTGATAGGTCACAACGGGGCTGGCAAAAGTACGTTCTTGAAAACTATAGCCGGTCTCTACCCTATATCATCTGGGACATTAAAAGTGACGGGTACCG   1080
Rev1Δwzm          TAGCGCGGGGCGAACGCGTTGCCCTGATAGGTCACAACGGGGCTGGCAAAAGTACGTTCTTGAAAACTATAGCCGGTCTCTACCCTATATCATCTGGGACATTAAAAGTGACGGGTACCG   438
Rev1Δwzt          TAGCGCGGGGCGAACGCGTTGCCCTGATAGGTCACA------------------------------------------------------------------------------------   997
Rev1ΔwzmΔwzt      TAGCGCGGGGCGAACGCGTTGCCCTGATAGGTCACA------------------------------------------------------------------------------------   355

Rev1              TAAGATCCCTGTTCGATATTGGTCTTGGGTTTGAGCCTGATGCAACTGGCCGTGAGAATATTCTTTACCGTGGGTTGCTTCTCGGACTAACGCCACGTTTCATGCGAGAGATCGAGGATG   1200
Rev1Δwzm          TAAGATCCCTGTTCGATATTGGTCTTGGGTTTGAGCCTGATGCAACTGGCCGTGAGAATATTCTTTACCGTGGGTTGCTTCTCGGACTAACGCCACGTTTCATGCGAGAGATCGAGGATG   558
Rev1Δwzt          ------------------------------------------------------------------------------------------------------------------------   997
Rev1ΔwzmΔwzt      ------------------------------------------------------------------------------------------------------------------------   355

Rev1              AGATCATCGAGTTCGCGGATCTCGGCGATTTTATCGATTATCCAATCAAAACTTATTCTGCCGGCATGCAAGTTCGGCTCGCCTTCGCGATTTCGACAGCAGTCGACGGCGACATACTCC   1320
Rev1Δwzm          AGATCATCGAGTTCGCGGATCTCGGCGATTTTATCGATTATCCAATCAAAACTTATTCTGCCGGCATGCAAGTTCGGCTCGCCTTCGCGATTTCGACAGCAGTCGACGGCGACATACTCC   678
Rev1Δwzt          ------------------------------------------------------------------------------------------------------------------------   997
Rev1ΔwzmΔwzt      ------------------------------------------------------------------------------------------------------------------------   355

Rev1              TTCTAGACGAAGTTATAGGTGCAGGTGATGCGGCATTCATGACTAAGGCGAAGGCCCGCATAATGAATATGGTCGAGAAGGCTGAGATAATGGTTCTAGCAAGCCATGACCTTGCGAACG   1440
Rev1Δwzm          TTCTAGACGAAGTTATAGGTGCAGGTGATGCGGCATTCATGACTAAGGCGAAGGCCCGCATAATGAATATGGTCGAGAAGGCTGAGATAATGGTTCTAGCAAGCCATGACCTTGCGAACG   798
Rev1Δwzt          ---------------------------------------------------------------------------------------------------CAAGCCATGACCTTGCGAACG   1017
Rev1ΔwzmΔwzt      ---------------------------------------------------------------------------------------------------CAAGCCATGACCTTGCGAACG   375

Rev1              TCCGTCAGCTTTGCACACGAGCATTGGTTTTCAAAGCCGGCACAATTGCATTTGATGGCAGGGTAGAAGACGCGATTTCCTTCTATAACTCGGGAATGGGAGCTATAGCATGA   1553
Rev1Δwzm          TCCGTCAGCTTTGCACACGAGCATTGGTTTTCAAAGCCGGCACAATTGCATTTGATGGCAGGGTAGAAGACGCGATTTCCTTCTATAACTCGGGAATGGGAGCTTTAACATGA   911
Rev1Δwzt          TCCGTCAGCTTTGCACACGAGCATTGGTTTTCAAAGCCGGCACAATTGCATTTGATGGCAGGGTAGAAGACGCGATTTCCTTCTATAACTCGGGAATGGGAGCTATAGCATGA   1130
Rev1ΔwzmΔwzt      TCCGTCAGCTTTGCACACGAGCATTGGTTTTCAAAGCCGGCACAATTGCATTTGATGGCAGGGTAGAAGACGCGATTTCCTTCTATAACTCGGGAATGGGAGCTATAGCATGA   488
```

Sequencing was performed with F7 *wzm* and R7 *wzt* pair of primers; ATG start and TGA stop codons are underlined.
