# Supplementary material for: Brucella melitensis Wzm/Wzt System: Changes in the Bacterial Envelope Lead to Improved Rev1Δwzm Vaccine Properties
Source: Front Microbiol. 2022 Jul 4;13:908495. doi: 10.3389/fmicb.2022.908495 (PMC9306315; doi:10.3389/fmicb.2022.908495)
Supplement: Supplementary file 6 [file Table_4.pdf]

Supplementary Table 4. Primers used for qPCR

Gene		Sequence (5’→3’) Forward	Reverse	Source/Reference
BMEI1671	*IF-1*	ttacggaactgctgcccaat	actagaaccttgtcaccggc	This work
BMEI1413	*gmd*	cagccgaggtggaaactctt	cagcaaattctcacgagccg	
BMEI1414	*per*	caagttagctggattgccgc	ggctctctgtgttccgagtt	
BMEI1404	*wbkA*	gttgaagggagcgattccga	gaagcgatctgcaagccaac	
BMEI1417	*wbkB*	agattcaggcgctccaagac	acagaagccggaatcgtcag	
BMEI1418	*wbkC*	tcggttgcgtgggtgattat	tgacttcctctaagcgcagc	
BMEI1427	*wbkD*	gacgcatgaagacatgacgc	gccagatcgcggattttcac	
BMEI1393	*wbkE*	cacttggcttggacgagtct	cccatccccgacaatgacaa	
BMEI1426	*wbkF*	ctgccaaaccttctgcccta	gccgagtaatgcgataccca	
BMEI0998	*wboA*	ggaaattgttgcgggtctcg	cgcagcaaaagttccgttgt	
BMEI0997	*wboB*	aatggctttctttgcgctcg	cgataagagcggtgaaggct	
BMEI1415	*wzm*	atgtctggaaggtacgccac	acgaaagacagtagcagcgt	
BMEI1416	*wzt*	cctgataggtcacaacgggg	tcaggctcaaacccaagacc	
BMEI1837	*cgs*	gccgatcagaaacaggcg	ttcccactggtgccttgc	Mirabella et al., 2013
BMEI0984	*cgt*	caatgtttcggtggtgcaga	tttcgaggttcttggcgtaatc	
