# Supplementary material for: Brucella melitensis Wzm/Wzt System: Changes in the Bacterial Envelope Lead to Improved Rev1Δwzm Vaccine Properties
Source: Front Microbiol. 2022 Jul 4;13:908495. doi: 10.3389/fmicb.2022.908495 (PMC9306315; doi:10.3389/fmicb.2022.908495)
Supplement: Supplementary file 7 [file Table_5.pdf]

Supplementary Table 5. Phenotyping of Rev1 *wzm*/*wzt* mutants by standard typing methods (Alton et al., 1989)

Strain	10% CO_2 requirement	Catalase/oxidase/urease tests	Acriflavine agglutination/ crystal violet	Growth* with/without 10% CO_2							
				Antibiotics		Thionine			Fuchsine		Safranin O
				P_5	$Str_{2.5}$	10	20	40	10	20	100
Rev1	No	+/+/+	-/smooth	-/-	+/+	+/+	+/-	-/-	+/+	+/+	+/-
Rev1Δ*wzm*	No	+/+/+	+/rough	-/-	+/+	+/+	+/-	-/-	+/+	+/+	-/-
Rev1Δ*wzt*	No	+/+/+	+/rough	-/-	+/+	+/+	+/-	-/-	+/+	+/+	-/-
Rev1Δ*wzm*Δ*wzt*	No	+/+/+	+/rough	-/-	+/+	+/+	+/-	-/-	+/+	+/+	-/-

+: positive reaction or growth; -: negative reaction or absence of growth. * Growth in BAB with antibiotics or in BAB-S with dyes; P_5: penicillin G 5 IU/mL; $Str_{2.5}$: streptomycin 2.5 μg/mL; dyes in μg/mL.
