# Supplementary material for: Brucella melitensis Wzm/Wzt System: Changes in the Bacterial Envelope Lead to Improved Rev1Δwzm Vaccine Properties
Source: Front Microbiol. 2022 Jul 4;13:908495. doi: 10.3389/fmicb.2022.908495 (PMC9306315; doi:10.3389/fmicb.2022.908495)
Supplement: Supplementary file 8 [file Table_6.pdf]

Supplementary Table 6. Growth of Rev1 *wzm*/*wzt* mutants in presence of antibiotics and concentrations to which Rev1 is resistant

Atmosphere	Medium	Number of CFU/100 µL (mean±SD; n=3)			
		Rev1	Rev1Δ*wzm*	Rev1Δ*wzt*	Rev1Δ*wzm*Δ*wzt*
Normal	BAB	416±54	247±20	263±37	216±39
	BAB-S	415±55	237±22	247±24	210±30
	BAB-$PxB_{1.5}$	386±34	1±1 [a]	1.3±1.5 [a]	1±1 [a]
	BAB-S-$PxB_{1.5}$	362±17	9±1 [a]	14±5 [a]	11±5 [a]
	BAB-Col_4	358±30	161±21 [a]	152±6 [a]	116±3 [a]
	BAB-S-Col_4	358±30	85±34 [a]	70±33 [a]	37±10 [a]
	BAB-$Str_{2.5}$	349±33	74±10 [a]	94±10 [a]	16±11 [a]
	BAB-S-$Str_{2.5}$	377±52	223±6	204±7	186±11
10% CO_2	BAB	386±28	238±17	257±34	208±41
	BAB-S	390±38	228±36	239±8	198±16
	BAB-$PxB_{1.5}$	256±19[a]	0 [a]	1±1 [a]	0 [a]
	BAB-S-$PxB_{1.5}$	256±19 [a]	0 [a]	1±1 [a]	0 [a]
	BAB-Col_4	267±21 [a]	8±2 [a]	4±3 [a]	2±0 [a]
	BAB-S-Col_4	314±50 [a]	1.3±1.5 [a]	3±1 [a]	3±1 [a]
	BAB-$Str_{2.5}$	309±67	170±22 [a]	171±10 [a]	167±13 [a]
	BAB-S-$Str_{2.5}$	264±21 [a]	104±7 [a]	104±9 [a]	87±8 [a]

BAB: Blood Agar Base No. 2 plates; S: 5% calf serum supplement; $PxB_{1.5}$: polymyxin B 1.5 µg/mL; Col_4: colistin 4 µg/mL; $Str_{2.5}$: streptomycin 2.5 µg/mL.
Fisher's LSD test: [a]$p \leq 0.01$ vs. growth in BAB plates incubated in normal atmosphere (shaded in grey).
