# Supplementary material for: Brucella melitensis Wzm/Wzt System: Changes in the Bacterial Envelope Lead to Improved Rev1Δwzm Vaccine Properties
Source: Front Microbiol. 2022 Jul 4;13:908495. doi: 10.3389/fmicb.2022.908495 (PMC9306315; doi:10.3389/fmicb.2022.908495)
Supplement: Supplementary file 9 [file Table_7.pdf]

Supplementary Table 7. Susceptibility to the antimicrobials recommended for human brucellosis treatment

Strain	MIC/ MBC_{90} (µg/mL)				Bacterial survival (mean %) in solid medium (µg/mL)						
	Dx	Rf	Gm	Str	$Dx_{0.02}$	$Rf_{0.4}$	$Gm_{0.125}$	$Str_{2.5}$	$Dx_{0.02}$+ $Rf_{0.4}$	$Dx_{0.02}$+ $Gm_{0.125}$	$Dx_{0.02}$+ $Str_{2.5}$
Rev1	0.025/0.1	0.50/1.0	0.25/0.25	15/>15	62.8%	59.0%	82.9%	89.8%	11.7%	66.3%	55.0%
Rev1Δ*wzm*	0.025/0.1	0.25/1.0	0.25/0.25	3.8/7.5	54.2%	38.8% [a]	59.2%[a]	37.7% [a]	8.6%	21.2% [a]	0.4% [ab]
Rev1Δ*wzt*	0.025/0.1	0.25/1.0	0.25/0.25	3.8/7.5	56.5%	39.6% [a]	60.0%[a]	39.7% [a]	7.8%	22.8% [a]	0.5% [ab]
Rev1Δ*wzm*Δ*wzt*	0.025/0.1	0.25/1.0	0.25/0.25	3.8/7.5	54.7%	40.9% [a]	61.1%[a]	41.9% [a]	9.0%	23.7% [a]	0.4% [ab]
E. coli K12	1.6/3.2	>16/>16	0.25/0.50	15/>15	ND	ND	ND	ND	ND	ND	ND

MIC: minimum inhibitory concentration preventing visible bacterial growth; MBC_{90}: minimum bactericidal concentration killing 90% of bacteria. Dx: doxycycline; Rf: rifampicin; Gm: gentamicin; Str: streptomycin. Each experiment was performed by triplicate. ND: not determined; Fisher's LSD test: [a]$p \leq 0.001$ vs. Rev1, [b]$p \leq 0.05$ vs. the other treatments
